# HSV-1 origin binding protein UL9 forms intermolecular DNA tethers and intramolecular DNA loops

**DOI:** 10.1038/s41467-026-74361-w

**Published:** 2026-06-29

**Authors:** Anita F. Meier, Jan Vuckovic, Paul Girvan, Adam S. B. Jalal, Erin Cutts, Theodora Brophy, Benjamin Ambrose, David S. Rueda

**Affiliations:** 1 Section of Virology, Dept. Infectious Disease, Faculty of Medicine, Imperial College London, London, UK; 2 Single-Molecule Biophysics Group, MRC Laboratory of Medical Sciences, London, UK; 3 Present Address: School of Biosciences, The University of Sheffield, Sheffield, UK

**Keywords:** Single-molecule biophysics, Herpes virus, Origin firing

## Abstract

Herpesviruses are ubiquitous human pathogens, causing mild to severe symptoms ranging from cold sores to nasopharyngeal carcinoma. Even though replication of the dsDNA genome has been studied for decades, we still lack a complete molecular understanding of its mechanism. It has been previously proposed, but never shown directly, that the HSV-1 origin-binding protein UL9 interacts with two closely spaced sites within the oriS origin sequence, thereby mediating origin looping, which in turn facilitates replication initiation. Here, we use an array of single-molecule approaches to test this long-standing hypothesis directly. Surprisingly, the data show that UL9 does not efficiently loop oriS. However, we demonstrate that UL9 can form large DNA loops at non-origin sequences very efficiently, as well as tether two oriS DNA molecules intermolecularly. Contrary to the origin-bending hypothesis, our findings indicate that UL9 does not primarily loop oriS DNA but rather may play an alternative role in replication initiation, such as tethering two separate molecules to facilitate recombination.

## Abstract (web summary)

The mechanism of herpes simplex virus type 1 (HSV-1) genome replication remains not fully understood. Here, the authors find that the HSV-1 origin binding protein UL9 does not mediate DNA looping but instead efficiently tethers DNA inter- and intramolecularly.

## Introduction

Herpes simplex virus type 1 (HSV-1) is a widespread human pathogen with a global seroprevalence of ~65% (WHO and refs. ^1–3^). Initial HSV-1 infection mainly occurs in the oral mucosal tissue and leads to productive replication. After an active replication phase, HSV-1 retreats into the sensory neurons of the trigeminal ganglia to establish latency^4^. While most infections remain asymptomatic, the most common symptom induced by an HSV-1 infection is cold sores. HSV-1 infections can also lead to more severe symptoms such as meningitis, keratitis, and even blindness^5^. More recent studies have suggested HSV-1 as a causative agent of Alzheimer’s disease^6,7^. HSV-1 is one of nine human pathogenic herpesvirus species and, therefore, shares many features with other herpesviruses such as varicella-zoster virus, Kaposi sarcoma-associated herpesvirus, and Epstein-Barr virus^8^. Given the potentially, however rarely, severe outcomes (e.g., tumor formation) of herpesvirus infections in humans and animals, it is of great importance to understand their intricate replication mechanism.

The HSV-1 genome consists of a 152 kb linear dsDNA within the viral capsid, which has been found to take on a circular conformation within the host nucleus through an unknown recombination mechanism^9–13^. However, there is evidence suggesting that HSV-1 genome circularization may not occur during lytic replication and predominantly plays a role during latency^14^. One study found that the HSV-1 E3 ubiquitin ligase ICP0^15,16^ actively inhibits circularization during lytic infections^14^. ICP0 is well known to suppress cellular DNA damage response pathways and actively promote the degradation of DNA PK^17–22^, which suggests that cellular factors promote circularization of the HSV-1 genome during latency. Genome replication is believed to occur first in an origin-dependent phase, followed by an origin-independent phase^23^. Although original models proposed that origin dependent replication occurs by a theta (θ) amplification mechanism, no direct evidence for this mechanism has been found, and thus, other models have been proposed^24,25^. The origin-independent phase has been suggested to occur in a rolling-circle amplification (RCA) mechanism. The product of RCA is expected to be large head-to-tail concatemers, which have been observed for HSV-1 replication^26,27^. However, such concatemers could also arise from a recombination-dependent mechanism^27–29^.

**Fig. 1:**
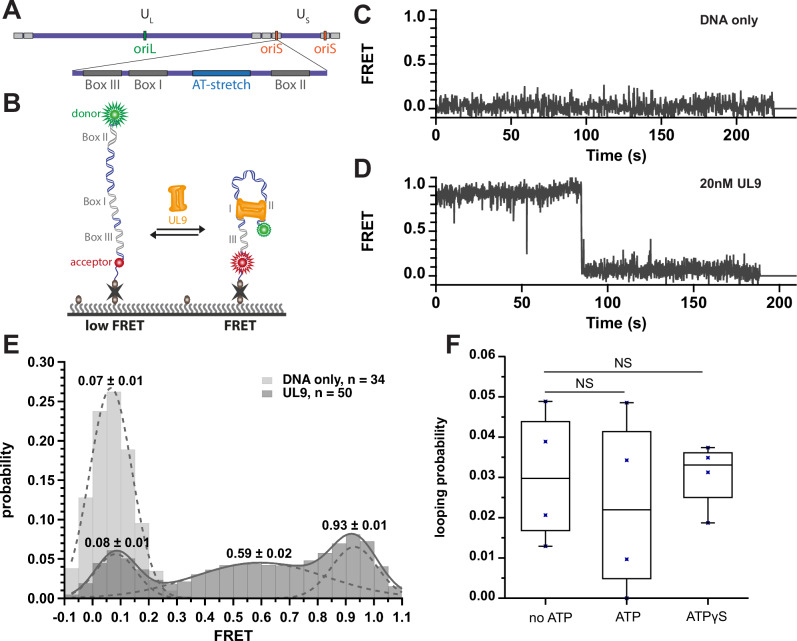
UL9 mediates origin looping at low efficiency. **A** Schematic representation of the HSV-1 genome structure, including the unique long (U_L_) and unique short (U_S_) sections (violet) as well as the flanking repeat regions (grey) and the origin sequences (ori_S_ (orange) and ori_L_ (green)). The different UL9 binding sites within oriS (Box I, Box II, and Box III (grey boxes) and AT-stretch (blue box)) are indicated. **B** Depiction of the smFRET looping assay, including a donor (Cy3, green) and acceptor (Cy5, red) labeled template DNA (grey/blue) tethered to the microscope slide (dark grey) through biotin-neutravidin (oval biotin, star-shaped neutravidin) interaction on a PEG-coated surface (grey “S”). **C** Example of an oriS-DNA only control FRET efficiency trace. **D** Example FRET efficiency trace of an oriS-DNA in the presence of 20 nM UL9. **E** Histogram of FRET efficiencies in the absence (light grey) or presence of 20 nM UL9 (dark grey). Molecules showing no FRET were not included. In the presence of UL9 (dark grey), 97% of trajectories behaved as DNA-only and were not included to highlight the distribution of looping molecules (3%). The data was fit to Gaussian distributions (dashed lines) with the center of each distribution and the overall fit (solid line) indicated. Numbers of individual FRET traces are indicated (*n*). **F** Boxplot of fractions of traces showing FRET divided by the total number of traces in the presence of 20 nM UL9 only (0.03 ± 0.02 SD, median = 0.03, Q1 = 0.02, Q3 = 0.04, min = 0.01, max = 0.05, *n* = 892) and with 1 mM ATP (0.02 ± 0.02 SD, median = 0.02, Q1 = 0.004, Q3 = 0.04, min = 0, max = 0.05, *n* = 523, *p* = 0.62) or ATPγS (0.03 ± 0.01 SD, median =, Q1 = 0.02, Q3 = 0.04, min = 0.02, max = 0.04, *n* = 706, *p* = 0.98). Blue dots represent data from independent experiments. Two sample *t*-test was performed with NS for *p* ≥ 0.05. Source data are provided as a Source Data file.

The HSV-1 genome can be divided into a unique long (U_L_) and a unique short (U_S_) section, flanked by repeat regions, and contains three origins of replication (Fig. 1A). The two sections of the genome have been shown to exist inverted to each other giving rise to four isomers^30–32^. These isomers are equally likely and arise upon genome replication. Thus, recombination seems to be an inherent part of HSV-1 replication. The two oriS replication origins are located within the repeat regions flanking U_S_, whereas the third one, oriL, is located within the unique region of U_L_.

HSV-1 encodes seven proteins essential for genome replication^23,33^. These are the origin-binding protein UL9, the single-stranded DNA-binding protein ICP8, the helicase-primase complex (UL5/UL8/UL52), and the viral polymerase and its processivity factor (UL30/UL42). UL9 has been shown to mediate replication initiation and to be required only within the first 6 h after infection^34,35^. However, how UL9 initiates HSV-1 genome replication remains largely unknown. UL9 is a superfamily II helicase and requires a 3′ single-stranded DNA (ssDNA) overhang to mediate strand separation^36–38^. Therefore, the UL9 helicase activity alone cannot mediate unwinding of the origins, which are located in dsDNA regions of the genome. It is possible that active transcription by RNA polymerase provides access to the origins. Although mutants in the helicase motifs fail to support HSV-1 replication^39,40^, the precise role of UL9 during replication initiation remains unclear. To investigate how UL9 interacts with DNA, we used single-molecule microscopy.

The oriS origin contains two high-affinity UL9 binding sites, Box I and Box II, that are separated by an 18 bp AT-rich sequence (Fig. 1A)^41,42^. Adjacent to Box I, oriS also contains an additional low-affinity binding site called Box III, which is palindromic to Box I (Fig. 1A). It has been proposed that UL9 mediates strand separation through simultaneous binding to the two oriS high-affinity binding sites (Box I and Box II), thereby looping and distorting the intervening AT-rich DNA structure^24,25,43^. In turn, this would facilitate strand separation and enable other replication factors to access the ssDNA and subsequent replisome assembly.

However, this long-standing “looping” model has never been tested directly. To this aim, we turned to single-molecule approaches, which eliminate ensemble averaging, thereby enabling us to image conformational dynamics in real-time. Importantly, force spectroscopy (optical tweezers) allows us to directly manipulate DNA molecules and measure changes in force, enabling us to gain a detailed understanding of how UL9 interacts with DNA.

Our data show that UL9 can induce oriS bending but very inefficiently. Unexpectedly, UL9 can induce loop formation very efficiently on large DNA templates independent of the origin sequence. We also found that UL9 has only a weak preference for the origin sequence and binds stably to other sequences in mechanically stretched DNA. Furthermore, we show that UL9 tethers dsDNA intermolecularly, raising the possibility that UL9 might play a role during recombination. Recombination has been proposed to play an intrinsic role during HSV-1 genome replication^28,29,44,45^, supported by previous observations of the high amount of recombination of HSV-1 genomes in cell cultures and in vivo^31,46–50^. Overall, our results point to a role of UL9 during recombination but do not exclude the possibility of oriS looping-mediated initiation.

## Results

### UL9 mediates oriS bending very inefficiently

To test the looping hypothesis, we surface-immobilized a 72 bp double-stranded DNA (dsDNA) template containing the oriS sequence with all three UL9 binding sites (Box I, Box II, and Box III, Fig. 1A) to a passivated microscope slide (Fig. 1B). The oriS sequence is flanked by a FRET donor (Cy3) and acceptor (Cy5) (see “Methods”) to measure the end-to-end distance of the DNA. We expect a long distance (low FRET) for the DNA alone and a short distance (mid to high FRET) upon loop formation. In the absence of UL9, we only observe trajectories at no ( ~0 FRET efficiency) or low FRET ( ~0.07 FRET efficiency, Fig. 1E and Supplementary Fig. 1A), confirming that the DNA is extended (Fig. 1C and E and Supplementary Fig. 1C). Addition of UL9 results in no apparent FRET efficiency increase for ~97% of observed molecules (*N* = 892, Supplementary Fig. 1B). Only a small fraction ( ~3%, Fig. 1F) exhibits FRET increases between 0.3 and 1 (Fig. 1D–F and Supplementary Fig. 1B and D), consistent with UL9-induced DNA looping. Addition of ATP, or non-hydrolyzable ATP analog (ATPγS), did not increase the fraction of molecules exhibiting mid to high FRET ( ~3%, Fig. 1F).

To confirm that UL9 binds the oriS DNA, we performed electrophoretic mobility shift assays (EMSA) and reveal an apparent *K*_1/2_ = 13 ± 1 nM and *n* = 1.9 ± 0.2 (Supplementary Fig. 2A–C).

**Fig. 2:**
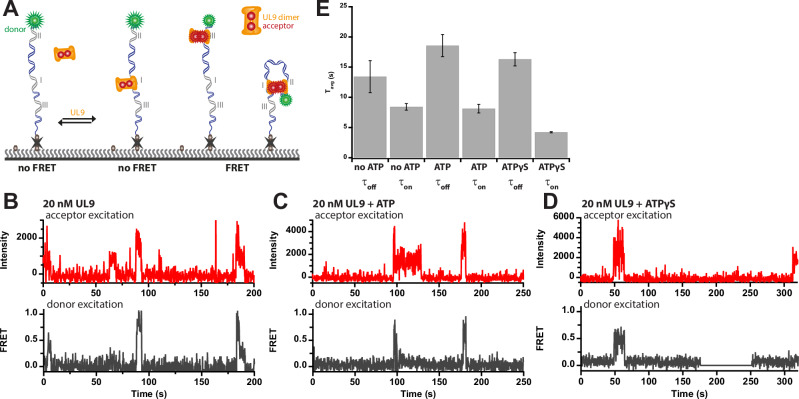
Interaction of UL9-atto647N with oriS-DNA. **A** Depiction of the UL9-atto647N binding assay, including a donor (Cy3, green) labeled template DNA (grey/blue) tethered to the microscope slide (dark grey) through biotin-neutravidin (oval biotin, star-shaped neutravidin) interaction on a PEG-coated surface (grey “S”) and acceptor (atto647N, red)- labeled UL9 dimer (orange). Example traces under acceptor (red, top row) or donor (FRET efficiency, grey, bottom row) in the presence of 20 nM UL9 in the absence (**B**) or presence of 1 mM ATP (**C**) or 1 mM ATPγS (**D**). **E** Bar plot of average dwell times (*τ*_avg_) of UL9-atto647N on the oriS template at a concentration of 20 nM in the absence (unbound (*τ*_off, avg_ = 13.4 ± 2.6 s, *n* = 1342), bound (*τ*_on, avg_ = 8.4 ± 0.5 s, *n* = 2404)), or presence of 1 mM ATP (unbound (*τ*_off, avg_ = 18.6 ± 1.8 s, *n* = 1714), bound (*τ*_on, avg_ = 8.1 ± 0.7 s, *n* = 1394)) or 1 mM ATPγS (unbound (*τ*_off, avg_ = 16.3 ± 1.1 s, *n* = 1712), bound (*τ*_on, avg_ = 4.2 ± 0.1 s, *n* = 1918)). Averages are extracted from the dwell time analysis shown in Supplementary Fig. 2D–F. Error bars indicate the highest observed error ratio (see Source Data). *n* = number of transitions. Source data are provided as a Source Data file.

To asses if UL9 also binds to the oriS substrate in our TIRF assay, we visualized UL9 binding by labeling its C-terminus with an atto647N (a FRET acceptor) fluorophore (UL9-atto647N) through a ybbR-tag^51,52^, and the surface-immobilized DNA with Cy3 only (FRET donor)(Fig. 2A). Using Alternating Laser Excitation (ALEX)^53^, we can simultaneously monitor UL9 binding by direct acceptor excitation (Fig. 2B–D, red) and approximate binding location by FRET (Fig. 2B–D, grey). In the absence of UL9-atto647N (DNA only), we clearly detect donor signal, corresponding to the immobilized Cy3 DNA, but no acceptor signal (Supplementary Fig. 2G). The fluorescence trajectories show that the majority of molecules (79 ± 9%, *n* = 316) bind UL9 readily, even in the absence of ATP or ATPγS. We frequently observed two photobleaching steps (Fig. 2B, C and Supplementary Fig. 2H–J, red), indicating that UL9 binds the oriS template as a dimer, as expected^54,55^. Based on the dwell times in the bound and unbound states, we quantify the binding kinetics of UL9 to the oriS DNA. The resulting dwell time histograms (Supplementary Fig. 2D–F) reveal biphasic dissociation kinetics with a short (*τ*_on,1_ = 1.0 ± 0.1 s) and a long dissociation time (*τ*_on,2_ = 9.7 ± 0.4 s) (Supplementary Fig. 2D). These two bound times likely correspond to two binding modes. One possibility is that UL9 dissociates from one of the Box sequences (slow, *τ*_on,2_) or the non-specific intervening DNA sequences (fast, *τ*_on,1_). Alternatively, UL9 could bind in two different orientations or conformations with different binding stabilities. The unbound dwell time histogram also shows biphasic binding kinetics with a short (*τ*_off,1_ = 3.0 ± 0.2 s) and a long (*τ*_off,2_ = 25.6 ± 5.0 s) binding time, consistent with the idea that UL9 can bind in two different modes. This assay revealed an apparent *K*_*d*_ of 31.9 ± 0.3 nM for UL9-atto647N in the absence of ATP. Addition of ATP did not considerably change the average dwell times, whereas addition of non-hydrolyzable ATPγS decreases the average bound time (τ_on,avg_) by approximately twofold while the average unbound time (τ_off,avg_) remains largely unchanged (Fig. 2E and Supplementary Fig. 2D–F), indicating that ATP binding but not hydrolysis induces a conformational change that destabilizes the bound complex.

Overall, our results indicate that UL9 rarely loops the oriS DNA, in accordance with the initial single-molecule FRET (smFRET) experiments (Fig. 1), raising the interesting possibility that UL9 cannot effectively bend DNAs shorter than the persistence length (*L*_*p*_ ~150 bp) and may have a different function in HSV-1 replication initiation.

### UL9 efficiently induces oriS-independent loops on long DNA

**Fig. 3:**
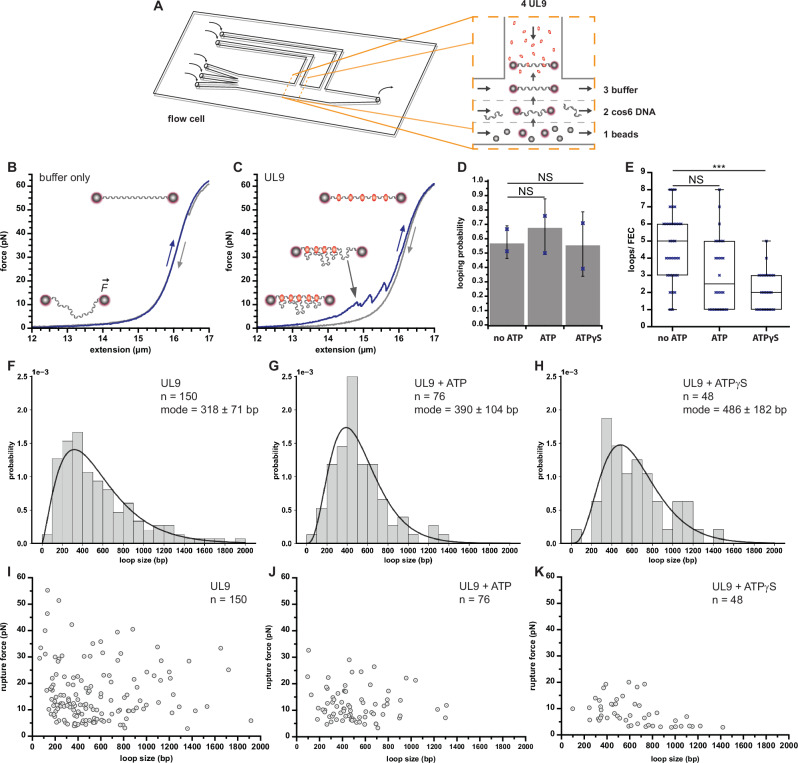
UL9 efficiently induces loops into large DNA substrates. **A** Schematic representation of the optical tweezer flow cell (black/grey), including a zoom-in (orange) of the channels. Arrows from left to right (and down) indicate the liquid flow direction. DNA (grey lines), polystyrene beads (grey spheres), optical tweezer location (red halo), and UL9 (orange ovals) are indicated. Upwards pointing arrows indicate the direction of the optical tweezers during an experiment. Force extension curves (FECs) of the HSV-1-derived cos6 template in buffer only (**B**) or in the presence of 10 nM UL9-atto647N (**C**) with depictions of the anticipated DNA conformation. Extension data is shown in blue and relaxation in grey. **D** Bar plot showing fractions of rip-containing FECs in the presence of 10 nM UL9-atto647N using cos6 without ATP (0.57 ± 0.11 SD, *n* = 60), with 1 mM ATP (0.67 ± 0.19 SD, *n* = 43, *p* = 0.82), or with 1 mM ATPγS (0.55 ± 0.22 SD, *n* = 47, *p* = 0.84). *n* = number of total FECs. Data for each condition was collected on two separated days. The dataset from one day was considered one experiment. **E** Boxplot showing number of rips per FEC in the presence of 10 nM UL9 in the absence (4.7 ± 2 SD, median = 5, Q1 = 3, Q3 = 6, min = 1, max = 8, *n* = 33) or presence of 1 mM ATP (3.2 ± 2.1 SD, median = 2.5, Q1 = 1, Q3 = 5, min = 1, max = 8, *n* = 24, *p* = 0.82) or 1 mM ATPγS (2.2 ± 1.1 SD, median = 2, Q1 = 1, Q3 = 3, min = 1, max = 5, *n* = 22, *p* = 0.000002). *n* = number of FECs. Data from individual FECs are indicated as blue dots. Two sample *t*-test was performed for (**D**, **E**) with NS for p ≥ 0.05, * for *p* ≤ 0.05, ** for *p* ≤ 0.01, and *** for *p* ≤ 0.001. Histogram of loop sizes in the presence of 10 nM UL9 in the absence (**F**), presence of 1 mM ATP (**G**), or 1 mM ATPγS (**H**) using cos6 DNA. *n* = number of loops. Gamma distribution fit is shown as grey line. Scatter plots showing the correlation of loop size versus rupture force on cos6 DNA in the presence of 10 nM UL9 in the absence of ATP (**I**), presence of 1 mM ATP (**J**), or 1 mM ATPγS (**K**), *n* = number of loops. Source data are provided as a Source Data file.

To test this hypothesis, we turned to optical tweezers experiments, which enable us to observe looping on long DNA substrates^56,57^. To this aim, we prepared a DNA substrate (cos6, 47.6 kb, see “Methods”) encoding about one third of the HSV-1 genome and containing a single origin of replication (oriS). The linearized and biotinylated cos6-DNA was optically tethered between two streptavidin-coated beads (Fig. 3A) and incubated in the absence and presence of UL9 near zero force for 5–10 s before recording force-extension curves (FECs).

In the absence of UL9, the cos6 DNA substrate exhibits a characteristic FEC that follows the extensible worm-like chain (eWLC) model, as expected (Fig. 3B and Supplementary Fig. 3A, blue). After stretching, decreasing the bead-to-bead distance allows the DNA to re-form into its original structure and follow the same FEC (Fig. 3B and Supplementary Fig. 3A, grey). In the presence of UL9, however, we observe numerous rips in the FECs (Fig. 3C and Supplementary Fig. 3B), indicating the formation of shorter contour length (*L*_*c*_) DNA likely due to UL9 looping the intervening DNA^58^, consistent with previous electron microscopy experiments^59^. We observed an average of 4.7 ± 2.0 (*n* = 33) loops per molecule in the absence of ATP, 3.2 ± 2.1 (*n* = 24) loops per molecule in the presence of ATP and 2.2 ± 1.1 (*n* = 22) in the presence of ATPγS (Fig. 3E and Supplementary Fig. 3C, D), indicating that ATP binding but not hydrolysis leads to loop destabilization.

Since the cos6 DNA template contains a single origin of replication, the presence of multiple rips in a single DNA pull (FEC) suggests that UL9 can induce oriS-independent loops within the cos6 DNA. Interestingly, looping of the cos6 DNA is very efficient ( ~ 60%, Fig. 3D) and looping efficiency does not seem to be affected by ATP binding or hydrolysis.

Next, we assessed the loop-size distribution (Fig. 3F–H), which can be fit to a gamma distribution as previously described^60–63^, defined by a scale (l_0_) and a shape (*n*) factor (see Methods). The distribution profile arises from the fact that loops smaller than the persistence length of dsDNA ( ~ 150 bp) are unlikely to occur, while large loops are entropically unfavorable^64^. In the absence of ATP, the most probable loop length is 318 ± 71 bp, reaching maximum loop sizes of ~2 kbp (Fig. 3F and I). Addition of ATP or the non-hydrolyzable analogue (ATPγS) results in longer most-probable loop sizes (390 ± 104 bp and 486 ± 182 bp, respectively) but shorter maximum loop sizes ( ~ 1.5 kbp, Fig. 3G, H, J, and K). This result is in line with our earlier observation that the addition of ATP or ATPγS reduces the number of loops per molecule (Fig. 3E), since fewer longer loops can fit in a given length of DNA template^62^. In agreement with this, the shape factor in the absence of ATP (*n* = 2.4 ± 0.3) is significantly lower than in the presence of ATP (4.0 ± 0.6) or ATPγS (4.4 ± 0.9), indicating that the addition of ATP or ATPγS restricts small loop formation. Along the same lines, the scale factor decreases in the presence of ATP or ATPγS from *l*_0_ = 225 ± 27 bp (no ATP) to 128 ± 21 bp and 143 ± 30 bp, respectively, indicating less variability in loop size. Overall, both the increase in n and the decrease in *l*_0_ indicate that the addition of ATP or ATPγS leads to more restrictive loop formation. The fact that ATP and ATPγS result in similar behaviors further suggests that ATP binding, but not hydrolysis, restricts loop formation. To confirm that the observed activities are specific to UL9 and not due to a contaminating DNA-binding protein, we purified and characterised an ATP-hydrolysis mutant (UL9 E175A)^39,65^ (Supplementary Fig. 8). Microscale thermophoresis (MST) confirms that this mutant cannot bind ATP (Supplementary Fig. 3I). In the presence of ATP, the optical tweezers data show that the mutant induces 4.8 ± 2.2 (*n* = 17) loops per molecule (Supplementary Fig. 3F), comparable to wildtype (wt) UL9 in the absence of ATP (4.7 ± 2.0, *n* = 33, Fig. 3E) and with shorter loop-size distribution (215 ± 69 bp, *n* = 81, with ATP) than the wt in the presence of ATP or non-hydrolyzable analogue (Supplementary Fig. 3E–H).

UL9-induced loops rupture at forces clustered around 10 pN in the absence or presence of ATP or ATPγS (Fig. 3I–K) or using the E175A mutant (Supplementary Fig. 3H), with maximum rupture forces in the absence of ATP around 55 pN. However, addition of ATP or ATPγS reduces the maximum rupture force to below 35 pN, suggesting that ATP binding but not hydrolysis destabilizes UL9-induced loops. In the presence of ATP, the E175A mutant exhibits the highest rupture forces ( ~ 59 pN, Supplementary Fig. 3H), further supporting the hypothesis that ATP binding but not hydrolysis destabilizes UL9-induced DNA loops (Supplementary Fig. 3H). Taken together, our data show that UL9 efficiently induces large oriS-independent DNA loops, which are destabilized by ATP binding.

### UL9 shows oriS sequence preference mainly at low force

**Fig. 4:**
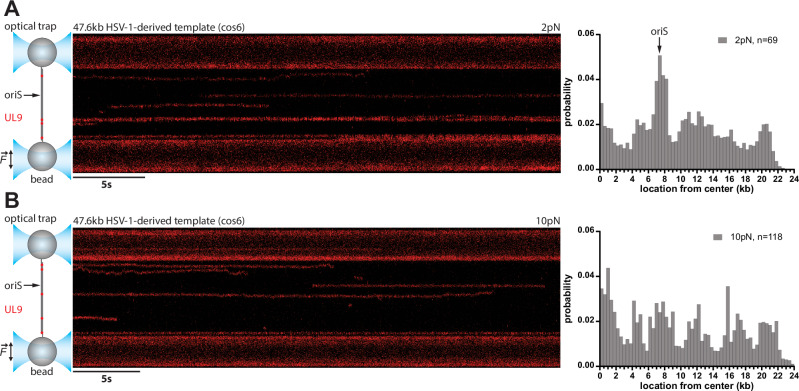
UL9 shows slight binding preference to the oriS origin sequence only at low forces. Schematic depiction of the bead (grey)-tethered cos6 DNA template (black lines) held by optical tweezers (blue) and interacting UL9-atto647N (red) (left). Example kymographs of UL9-atto647N binding (red traces) to different sites on the cos6 template DNA held at 2 pN (**A**) or 10 pN (**B**) are shown. Histograms of binding sites of UL9-atto647N to cos6 DNA plotted from the center of the DNA are shown on the right with DNA held at 2 pN (**A**) or 10 pN (**B**). *n* = number of UL9-atto647N traces. Location of oriS sequence is indicated (7.2 kb from the center). Source data are provided as a Source Data file.

Since UL9 appears to form oriS-independent loops, we sought to test whether UL9 has any sequence-specificity on the cos6 DNA, which contains a single oriS sequence located 7.2 kbp from the center of the linearized substrate (Fig. 4). To visualize UL9 binding, we used UL9-atto647N, such that binding appears as bright fluorescent signal on pre-stretched cos6 DNA at 2 pN force. The resulting kymographs (Fig. 4) show that UL9 binds throughout the DNA without noticeable free diffusion^66^, revealing several oriS-independent binding events. The DNA can be tethered in two possible orientations, making it challenging to determine the oriS position on each molecule. Therefore, to examine the spatial distribution of UL9 foci on the DNA, we plotted cumulative binding times as a function of radial distance from the mid-point of the DNA, as described in ref. ^67^. At low stretching force (2 pN), we see UL9 binding throughout cos6 DNA with a peak at the expected oriS location in the absence or presence of ATP (7.2 kbp, Fig. 4A and Supplementary Fig. 4A, E). However, this preference is lost at higher forces in the absence of ATP (> 5 pN, Fig. 4B and Supplementary Fig. 4). Interestingly, the binding preference for oriS seems to be more pronounced in the presence of ATP even at higher forces (Supplementary Fig. 4). Furthermore, cos6 also contains the high-affinity binding site^68^ within the UL9 ORF (“UL9 box”; TGTTCGCACT) at 11.2 kb from the middle, to which UL9 only seems to preferentially bind in the presence of ATP at 10 pN (Supplementary Fig. 4G). Nevertheless, we can clearly observe prolonged binding to non-origin sequences under all tested conditions. Using λ-DNA as (non-HSV-1 DNA) template, we can clearly see that UL9 also binds DNA that was not derived from HSV-1 (Supplementary Fig. 4I, J). Importantly, λ-DNA contains one copy of “CGTTCGCACT”, the Box I sequence^69,70^, located ~20.2 kb from the center. In parallel with other locations on the λ-DNA, UL9 exerts clear preference for the Box I sequence (Supplementary Fig. 4I, J). Unfortunately, the experimental resolution and the added difficulty of two possible DNA orientations, prevent the identification of additional binding sequences in a more accurate manner.

### UL9-induced loops are static

**Fig. 5:**
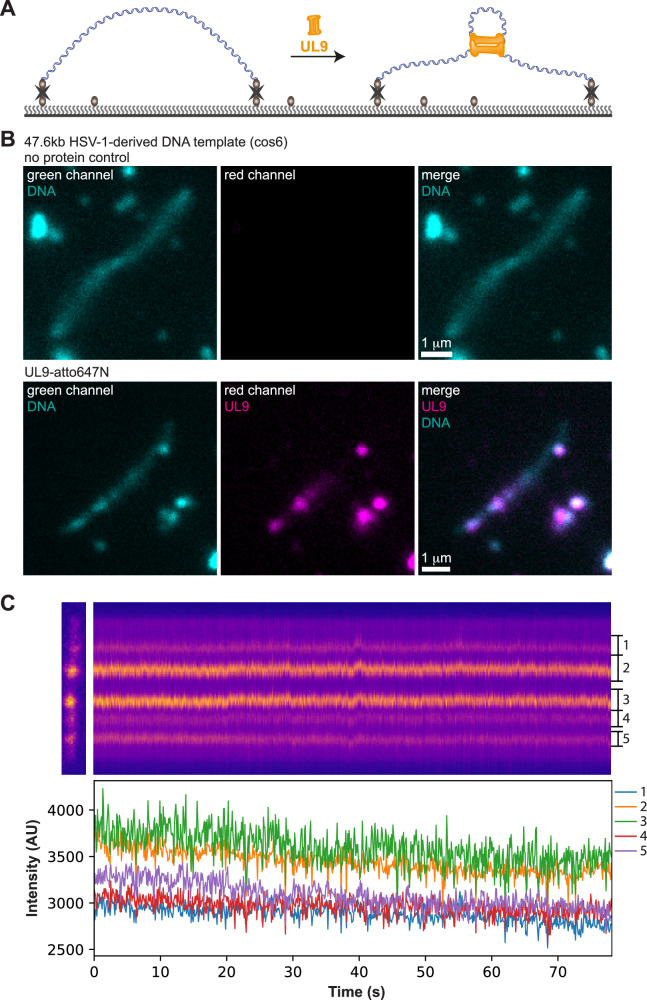
DNA loops induced by UL9 are static. **A** Schematic depiction of the experimental setup showing UL9 (orange) interacting with double-tethered template DNA (blue). Oval shapes indicate biotin, star shapes neutravidin on top of a PEG-layer (S-shaped), and the microscope slide (dark grey). DNA is shown in blue and UL9 in orange. **B** Example microscopy images of sytox orange labeled cos6 DNA (turquoise) in the absence (top row) or presence (bottom row) of 10 nM UL9-atto647N (purple). Data with atto647N-labeled UL9 was collected in one experiment. **C** Kymograph (top) of cos6 DNA in the presence of 20 nM UL9 with the loop-containing sections indicated on the right. Images were colored using the fire LUT to emphasize differences in intensity spanning the lowest (black) and highest (white) intensity value. Intensities of the individual loop sections are shown below. Intensity was determined by calculating the intensity per pixel within individual loop sections on the DNA over time. 6 independent experiments with double-tethered cos6 DNA and unlabeled UL9 were performed. Source data are provided as a Source Data file.

To assess the dynamics of UL9-induced loops, we used a TIRF-based assay with surface-tethered cos6 DNA on passivated slides (Fig. 5A), as described in refs. ^71,72^. In the absence of UL9, surface-tethered and sytox orange-stained DNA molecules show evenly distributed signal along the length of the DNA, indicating a lack of local compaction of the DNA (Fig. 5B and Supplementary Movie 1). In the presence of UL9-atto647N, we observed clear accumulation of fluorescence density at various spots on the template DNA, consistent with the idea that UL9 induces local DNA compaction (Fig. 5B, C and Supplementary Fig. 5A). Furthermore, the atto647N signal overlaps with the compacted high-intensity DNA spots (Fig. 5B and Supplementary Fig. 5A). None of the observed high-intensity spots (DNA loops) showed migration along the DNA, and the spot intensities remained relatively constant over time, indicating that UL9-induced loops are static (Fig. 5C). Addition of 4 nM UL9 led to an average loop number of 2.7 ± 1.3 (*n* = 59) per molecule in the absence of ATP, which did not change significantly (*p* = 0.157) in the presence of ATP (3.1 ± 1.3 loops/molecule, *n* = 34) (Supplementary Fig. 5E), in agreement with the optical tweezers data (Fig. 3). We observed a few cases (*n* = 8) of newly emerging loops and all of them were formed in a rapid stepwise manner (Supplementary Fig. 5B–D and Supplementary Movie 1). We also observed rapid stepwise disappearance of 4 loops. Some of the loops also seemed to spontaneously merge, forming higher-intensity structures (Supplementary Fig. 5C, D). In agreement with the optical tweezer experiments, we also observed HSV-1 DNA-independent looping using λ-DNA (Supplementary Fig. 5C, D). Together, these results suggest that UL9 forms DNA loops by rapidly tethering two distant sites on the template DNA and remains bound statically.

### UL9 tethers dsDNA in trans

**Fig. 6:**
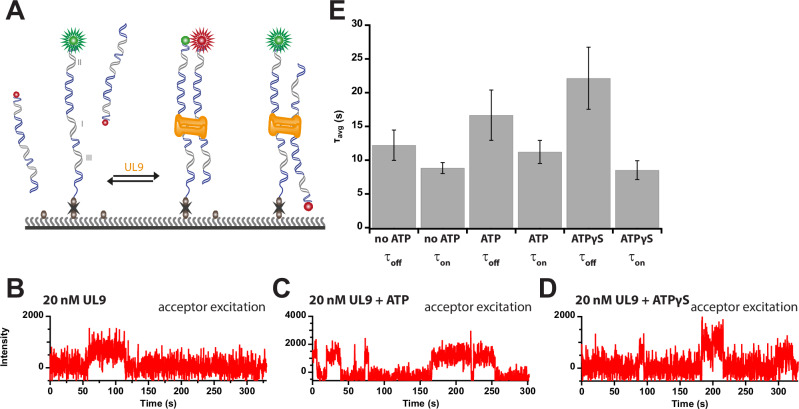
UL9 tethers dsDNA inter-molecularly. **A** Schematic of the experimental setup with donor Cy3- (donor, green) labeled DNA (blue/grey) tethered to the surface and Cy5- (acceptor, red) labeled DNA in solution. Oval shapes indicate biotin, star shapes neutravidin on top of a PEG-layer (S-shaped), and the microscope slide (dark grey). Example acceptor traces under direct acceptor excitation (red) in the presence of 20 nM UL9 without (**B**), with 1 mM ATP (**C**), or with 1 mM ATPγS (**D**). **E** Bar plot of average Cy5-DNA dwell times (*τ*_avg_) in the absence (*τ*_off, avg_ = 12.2 ± 2.2 s, *n* = 740, *τ*_on, avg_ = 8.8 ± 0.8 s, *n* = 740) or presence of 1 mM ATP (*τ*_off, avg_ = 16.7 ± 3.7 s, *n* = 695, *τ*_on, avg_ = 11.2 ± 1.7 s, *n* = 718) or 1 mM ATPγS (*τ*_off, avg_ = 22.1 ± 4.6 s, *n* = 662, *τ*_on, avg_ = 8.5 ± 1.4 s, *n* = 662) extracted from the dwell time analysis shown in Supplementary Fig. 6D–F. Error indicates the highest observed error ratio (see Source Data). *n* = number of transitions. Source data are provided as a Source Data file.

Given that UL9 can form DNA loops by tethering two distant binding sites, we reasoned that UL9 may also be able to tether two distinct DNA molecules in trans. To assess this, we turned to our TIRF set-up with a surface-immobilized Cy3-labeled oriS template (Fig. 6A) and a non-biotinylated Cy5-labeled oriS dsDNA in solution. Using ALEX, we can monitor interactions between the two DNAs by directly exciting the two dyes sequentially. In the absence of UL9, we never observed any Cy5-signal colocalizing with surface-immobilized Cy3-oriS templates, showing that the two DNAs do not interact with each other, as expected. However, in the presence of UL9, we found clear Cy5 signal colocalizing with the surface-immobilized Cy3-oriS DNA (Fig. 6B and Supplementary Fig. 6A), showing that UL9 can readily bring together two DNA molecules in trans. UL9-mediated intermolecular binding of DNA was about 10 times more likely (30.0 ± 6.9%, *n* = 329) than looping of the short oriS template ( ~3% Fig. 1F). The trajectories show that UL9 tethers the two DNAs transiently and repeatedly, reminiscent of the UL9 binding trajectories (Fig. 2). To quantify these trans-tethering kinetics, we performed dwell time analysis. In line with the UL9 binding experiments, we observed biphasic exponential decays with fast and slow bound times (Supplementary Fig. 6D–F). The resulting average bound and unbound half-lives (*τ*_on,avg_ and *τ*_off,avg_, Fig. 6E) are very similar to those obtained for UL9 binding, showing that UL9 captures the Cy5-oriS DNA in solution and brings it to the surface DNA. Surprisingly, the addition of ATP or ATPγS did not affect the half-lives significantly (Fig. 6B–E and Supplementary Fig. 6B–F). Taken together, these data show that UL9 can effectively tether two separate oriS molecules, raising the interesting possibility that UL9 may be involved in facilitating recombination.

## Discussion

A search for proteins that bind to the oriS sequence led to the identification of UL9, and subsequent footprinting analysis revealed the exact UL9 binding sites within Box I and Box II^43,73,74^. On its own, UL9 can unwind 100–200 bp^36,55^ and up to 2 kb in the presence of ICP8^38,75^. However, UL9 has been found to be unable to unwind blunt DNA substrates and requires a 3′ ssDNA overhang even in the presence of ICP8. Subsequent studies revealed that the spacing between Box I and Box II impacts the replication efficiency and found that a periodicity of 1 helical turn is crucial to maintain replication capacity^41^. This suggests that UL9 would need to interact with both sequences simultaneously and that this interaction has a fixed orientation requirement. Detailed enzymatic and chemical footprinting studies revealed that UL9 distorts the helix within the AT-rich region between Box I and Box II^43^. These results then led to the hypothesis that UL9 distorts the double helix by looping the oriS sequence. Given that these assays were all gel-based, DNA accessibility, distortion, and sequence requirements rather than direct DNA looping, were assessed. More direct evidence for UL9-induced DNA looping was provided by using electron microscopy^59^. Binding of UL9 to a short piece of DNA led to local DNA bending at an angle of 35 ± 15° if a single dimer was present, 86 ± 38° if a double dimer bound. On larger (2.6 kb) DNA substrates, they showed that UL9 induces large DNA loops with sizes ranging from a few hundred bp to above 1 kb. Interestingly, they found that the majority of the loops contained the oriS sequence, and therefore, loop formation was suggested to be oriS-dependent. However, given that the oriS sequence in their experimental setup was located very central within the template, the likelihood of such loops to contain the origin sequence is relatively high.

Here, we have used single-molecule microscopy to test the UL9-induced oriS-looping model. Unexpectedly, the data shows that UL9 does not efficiently loop oriS sequence, even in the presence of ATP or a non-hydrolyzable ATP analog (Fig. 1), despite binding the DNA tightly (Fig. 2). A possible explanation for the low efficiency of oriS looping is that surface tethering might influence the observed looping efficiency because of geometric or mechanical constraints of the short surface-immobilized DNA construct used in the smFRET assay. But we don’t think this is a likely explanation because (i) the binding affinities measured by EMSA and smFRET are comparable (apparent *K*_*d*_ = 131 ± 70 and 31.9 ± 0.3 nM, respectively, Supplementary Fig. 2), and (ii) loops shorter than 100 bp are not frequently observed in the flexible tethering geometry of the optical tweezers experiments (Fig. 3F–H). However, using optical tweezers and TIRF microscopy (Figs. 3 and 5), we found that UL9 very efficiently induces large DNA loops independent of the origin sequence or ATP. In fact, we found that ATP binding, but not hydrolysis, destabilizes loop formation (Fig. 3 and Supplementary Fig. 3). We observed that the UL9 E175A mutation leads to increased loop stability and smaller loop sizes when compared to wt UL9 in the absence of ATP (Supplementary Fig. 3E–H). The glutamate at position 175 is known to interact with Mg^2+^ and mutating it to alanine abrogates the UL9 ATPase activity^39,65^. We confirmed by MST (Supplementary Fig. 3I) that the E175A mutation prevents ATP binding. Thus, inhibition of ATP binding leads to stronger DNA tethering. As mentioned, UL9-induced formation of large dsDNA loops has previously been found by Makhov et al. ^59^ by using electron microscopy, but they observed that the loops were always surrounding the origin sequence. This previous finding suggested that initial UL9-mediated loop formation originated at the origin and then progressed further away from the origin. In contrast, we find that UL9-mediated loop formation is largely origin-independent (Fig. 4). Our TIRF experiments suggest that UL9-induced loops are overall very static and don’t migrate. We propose that UL9 induces loops by tethering two distant locations on the DNA rather than performing a loop-extrusion mechanism. This hypothesis is further supported by our observation that UL9 is able to tether dsDNA intermolecularly. If DNA looping would arise from loop extrusion, we wouldn’t expect UL9 to be able to capture DNA from solution and tether it to the immobilized DNA. Our finding is further supported by recent studies of Gustavsson et al., where they revealed the UL9 structure and clearly show intermolecular DNA tethering by UL9^76^. Even though UL9 has previously been shown to bind non-origin sequences^68^, we did not expect that UL9 would show stable binding to such a wide range of sequences on HSV-1-derived (cos6) or non-HSV-1 (λ) DNA. Furthermore, we only found a mild preference for the origin at low DNA stretching forces (Fig. 4A). This force-dependent sequence-specificity might be explained by the previously found interaction of UL9 with the major groove, which could be impaired by DNA distortions^77^. Importantly, the recently published structure of UL9 shows its sequence-specific interaction with the origin major groove through its RVKNL motif but also demonstrates an extensive network of interactions with the phosphate backbone, which are sequence-independent^76^. This further supports our finding of sequence-independent interaction of UL9 with DNA. Interestingly, the force-sensitivity of origin-binding specificity observed here could indicate the presence of a force-dependent mechanism of UL9 regulation. UL9 is only required during the first 6 h post-infection^34,35^ and is even inhibitory if over-expressed^78–80^. Forces applied during DNA replication, for example, could be acting as mechano-regulators of UL9 activity. Indeed, several polymerases have been shown to function in this force regime^81–85^.

Our findings, combined with previous observations^43^, indicate that the origin-binding protein UL9 can lead to origin DNA bending, which might lead to local DNA distortion and melting. However, UL9-mediated oriS-bending is highly inefficient and thereby limiting. If UL9-mediated DNA looping occurs in the cell at the same level as we’ve observed with our smFRET assay, this low efficiency might ensure that replication is only initiated at low levels during the first replication stage, thus preventing fork collision and DNA damage resulting from fork stalling. In contrast, UL9-dependent intra- and intermolecular DNA tethering of larger DNA templates suggests that UL9 might predominantly be involved in a different mechanism. These findings point to a possible role of UL9 during HSV-1-mediated DNA recombination. Within the capsid, the 152 kb HSV-1 genome is not circularized. Soon after infection and entry into the nucleus, the number of genome ends have been shown to decrease^11,13^, indicating that end recombination is taking place, which has previously been suggested to lead to circularization^9,10,86^, concatemerization^27,45^ or formation of branched DNA structures^87^. Interestingly, the newly replicated genomes were also found to contain inversions strongly suggesting a role of recombination during HSV-1 genome replication^27,31^. While cellular factors could be mediating recombination, previous studies suggest that recombination is mediated by viral factors such as the ssDNA binding protein ICP8 and the 5′ to 3′ exonuclease UL12^28,29,88,89^. UL12 was identified as a possible candidate involved in recombination based on its homology to the bacteriophage λ exonuclease Redα^44^. In bacteriophage λ, Redα and Redβ have been shown to be the only factors necessary to mediate homologous recombination (HR)^90–92^. Analogous to Redβ, the HSV-1 ssDNA binding protein ICP8 was shown to mediate ssDNA strand annealing^88,93–96^. Even though UL9 has so far not been suggested to play a role during HSV-1 genome recombination, it was previously shown that UL9 knock-down altered the recombination of adeno-associated virus genomes in an HSV-1 co-infection setup^97^. In conclusion, we show that UL9 efficiently and rapidly tethers origin- and non-origin dsDNA leading to the formation of static DNA loops with preferential sizes of about 300 to 500 bp and the ability to bend the origin at low efficiency.

## Methods

### Protein expression and purification

Sf9 insect cells were used to generate baculovirus stocks and High5 insect cells were used for protein expression. Sf9 were maintained in SF900-III SFM (ThermoFisher, 12658019) and HighFive in SFX insect cell media (cytiva, SH30278), both supplemented with penicillin/streptomycin in a shaking incubator at 27 °C. HSV-1 UL9 was PCR amplified from the pCM-UL9^98^ plasmid (kindly provided by C. Fraefel, UZH, Switzerland) (primers: AMg003 (CGGATCCAatgcctttcgtgggggg), AMg004 (AAGAGAATCtagggtgctaaagttcaccgcc)) into the pLIB vector containing a C-terminal 3C-ybbr-tev-strepII tag using Gibson cloning, resulting in the pLIB-UL9-ybbR-tev-strep plasmid. To make the UL9 E175A mutant, a point mutation was introduced into pLIB-UL9-ybbR-tev-strep at UL9 aa position 175 (E to A) and an additional silent mutation was introduced to delete the HincII restriction site using the NEB Q5 site-directed mutagenesis kit and primers AMg022 (CGTTCTGGACgcgGTTATGTCcACG) and AMg023 (AGGACGTCGTAGTTGTTC) in line with previous cloning approaches^39^. In order to generate a bacmid, pLIB-UL9-ybbR-tev-strep was transformed into DH10EMBacY *E. coli* for transposition and generation of a UL9-encoding bacmid. Purified bacmid was then transfected into Sf9 insect cells to generate P0 baculovirus. After 72 h, virus was harvested. For further amplification, the P0 UL9-baculovrius stock was passaged in SF9 cells before being used for expression in either Sf9 or HighFive cells. Cell pellets were resuspended in purification buffer (20 mM HEPES pH 7.5, 250 mM NaCl, 1 mM MgCl_2_, 1 mM DTT, 10% glycerol) supplemented with 2 EDTA-free protease inhibitor tablets (Roche) per 50 ml and 25U/ml of benzonase/nuclease (Merck) and lysed with a dounce homogeniser followed by 3 min (10 s on/10 s off) sonication at 20% amplitude on ice. Lysate was cleared by centrifugation followed by supernatant filtration through a 0.45 µm filter before being loaded onto a StrepTrap HP or StrepTrap XT column (cytiva), washed with purification buffer and eluted with purification buffer supplemented with 5 mM d-desthiobiotin (Merck) or 50 mM biotin (Merck) respectively. FPLC purification was performed on an Äkta pure (cytiva). Protein containing fractions were pooled, diluted twofold with buffer A (20 mM HEPES pH 7.5, 5% glycerol, 1 mM DTT), loaded onto HiTrap Heparin HP column (cytiva), washed with Buffer A with 200 mM NaCl, then eluted with a gradient up to 1 M NaCl. UL9-containing fractions (coming off with about 600 mM NaCl) were collected and then concentrated using 30 K MWCO spin-concentrators (Amicon Ultra, Merck). Unlabeled protein was aliquoted and snap-frozen after the heparin column using liquid nitrogen. For labeling, concentrated (ybbR-tagged) UL9 was incubated with Sfp transferase, 5–10× excess of high-performance liquid chromatography (HPLC)-purified CoA-conjugated atto647N dye, tev protease (NEB) and 10 mM MgCl_2_ in buffer over night at 4 °C or for 30 min at 20 °C. After centrifugation, supernatant was loaded onto a size exclusion column (Superdex 200 Increase 10/300 GL, cytiva) and fractionated with purification buffer. Protein-containing fractions were pooled and concentrated using a 30 K MWCO spin-concentrator (Amicon Ultra, Merck). Purification steps were assessed by performing SDS PAGE with small samples (10 - 15 μl)   of the fractions (4–15% or 4–20% precast gel, BIO-RAD) and staining the gels with coomassie blue (Coomassie InstantBlue, abcam) (Supplementary Fig. 7). Final protein concentrations were measured using a Qubit (Thermo Fisher Scientific) and labeling efficiency was assessed using absorbance (BioDrop). Labeling efficiency of UL9-atto647N was estimated to be ~10% for the initial protein batch and increased to ~50% for later batches by shortening the incubation time during the protein labeling step to prevent aggregation.

### Optical tweezers

Optical tweezer confocal microscopy experiments were performed on the commercially available Lumicks C-trap with integrated confocal microscopy and microfluidics. Data was acquired using the Lumicks Bluelake software. All channels of the microfluidics chip were first passivated with ~1 ml Pluronic F-127 (0.5% w/v in PBS) flown through at 1–2 bar. Bacterial stocks with the HSV-1 sequence containing cos6 cosmid^99^ were provided by C. Fraefel (UZH, Switzerland). After extraction using a QIAGEN MiniPrep kit, the cos6 cosmid was cut with the single-cutter XbaI (NEB), leaving a 4 nt overhang on each side. The overhang was filled in using a Klenow (exo−) polymerase (NEB) in the presence of dGTP, dTTP (NEB), biotin-14-dATP, and biotin-14-dCTP (Jena Bioscience), resulting in two biotins at each end^100^. λ-DNA ends (Thermo Scientific SD0011), which contain 12 nt ssDNA overhangs, were biotinylated in the same way and as previously described^56^. Biotinylated DNA was caught in the C-trap flow cell by optically trapping 4.5 μm streptavidin coated polystyrene beads (Spherotech) at 0.005% w/v in laminar flow. DNA integrity was verified before each experiment by generation of force-extension curves (FECs) from 0 to 55 pN in the buffer channel. For confocal imaging of atto647N, 638 nm emission wavelength and red filter 640 LP for emission was used. To record kymographs, DNA was stretched to the indicated force, then moved into the UL9-atto647N containing channel while maintaining the same bead-to-bead distance. Within the protein channel, fresh protein was flushed in ( ~ 0.3 bar for ~5–10 s), the flow was turned off and imaging started while maintaining the required force using force clamping. To record FECs in the presence of UL9, the double-tethered DNA molecule was moved into the protein channel. Then, fresh protein was flushed into the channel at low pressure (0.3 bar) for 5–10 s while the DNA template was held at no tension ( ~ 11 μm). FECs were recorded with 0.4 μm/s.

### DNA oligonucleotide purification and labelling

Custom DNA oligonucleotides (Table 1) were synthesized by Integrated DNA Technologies (IDT) and purified by extraction from denaturing 12.5% PAGE by cutting the appropriate band from the gel and soaking overnight in elution buffer (40 mM Tris-Cl, pH8, 480 mM sodium acetate, pH5.5, 0.5 mM EDTA) followed by ethanol/sodium acetate precipitation. For labelling, the C6-dT amino-modified oligonucleotide was added to monoreactive dye (Cytiva, Q13108 (Cy3) and Q15108 (Cy5)) in 100 mM sodium bicarbonate solution and incubated over night at 4 °C in the dark. On the next day, the labelled oligonucleotide was purified by high-performance liquid chromatography (HPLC) using an analytical C8-5 column. Purified oligonucleotides were dried using a speed-vac centrifuge.

**Table 1:** DNA substrate sequences used in the TIRF assays

Name	Description	Sequence (5′ to 3′)
AM057	Box I/ II/ III-containing 72 bp oriS template for looping and DNA capture experiments Labelled with Cy3 for looping and UL9-interaction assay and labelled with Cy5 for the capture assay	/5AmMC6T/ccagtgctcgcacttcgccctaataatatatatatattgggacgaagtgcgaacgcttcgcgttctcacttc
AM071	Complementary to AM057 with a ssDNA tail of 9 adenines Labelled with Cy5 for looping and unlabeled for the capture and UL9-interaction assays	/5Biosg/aaaaaaaaa/iAmMC6T/gaagtgagaacgcgaagcgttcgcacttcgtcccaatatatatatattattagggcgaagtgcgagcactgg
AM058	Complementary to AM057 (without tail) used in the capture assay	gaagtgagaacgcgaagcgttcgcacttcgtcccaatatatatatattattagggcgaagtgcgagcactgg

### Microscope slide passivation and flow chamber assembly

Quartz (for smFRET, UQC optics) or glass (for double-tethered DNA experiments, Menzel-Gläser, ThermoScientific) slides with custom-drilled holes and glass coverslips (24 × 50 mm, No. 1.5H, VWR) were coated with aminosilane (N-(2- Aminoethyl)-3-aminopropyltrimethoxysilane) then pegylated using methoxy-PEG-SVA (Mr = 5000, Laysan Bio, Inc.) containing 10% biotin-PEG-SVA (Mr = 5000, Laysan Bio, Inc.) in 100 mM sodium bicarbonate as described previously^101^. Following passivation, slides and coverslips were stored in nitrogen at −20 °C in the dark. Prior to use, slides and coverslips were equilibrated to room temperature. If necessary, tubing was glued into the holes in the microscope slides using epoxy and excess tubing cut off. The flow chambers were then assembled using double-sided adhesive tape or 0.12 mm thick double sided adhesive sheets (Grace Bio-Labs SecureSeal). Flow chambers were sealed with epoxy glue.

### smFRET experiments

Biotin-PEG-passivated home-built quartz flow chambers (see sections above for more details) were incubated with 0.1 mg/ml neutravidin in T50 (50 mM NaCl and 50 mM Tris-Cl (pH 7.5)) for 5 min, then blocked for 10 min with Pluronic F-127 (0.5% w/v in T50). PAGE-purified biotinylated and fluorescently labelled complementary oligonucleotides (see other sections for more details and Table 1) were annealed at a concentration of 1 µM in T50 by incubating at 95 °C for 45 s followed by 10–15 min annealing at room temperature and immobilized on the flow chamber at 5 pM and 2 min incubation. After DNA-immobilization, a further biotin (1.7 µM) blocking step in UL9 buffer (50 mM NaCl, 0.1 mg/ml BSA, 40 mM Tris-Cl (pH7.5), 1 mM MgCl_2_, 0.5 mM DTT, and 50 mM potassium glutamate) was performed. Imaging was performed in UL9 buffer supplemented with glucose-oxidase scavenger system (8 mg/ml D-glucose, 1 mg/ml oxidase, and 40 µg/ml catalase) in the presence or absence of 20 nM UL9-atto647N or 20 nM UL9 and 1 mM ATP or 1 mM ATPγS. For the DNA capture assay, Cy5-labelled (non-biotinylated) DNA was provided at 10 nM in solution. Data acquisition was performed on an Olympus IX-71 microscope equipped with a home-built prism-TIRF module. Excitation was provided by a 532 or a 637 nm laser. Fluorescent signal was collected through a 1.2 NA, 60× water objective (Olympus) and filtered through a dual bandpass filter (FF01-577/690-25, Semrock). The fluorescence was spectrally separated using an OptoSplit II (Cairn Research) to separate donor and acceptor emission. The donor and acceptor emission were further filtered through ET585/65M and ET700/75M (Chroma) bandpass filters, respectively. The donor and acceptor images were then projected side-by-side onto an EMCCD camera (Andor iXon Ultra897). Data was collected as raw movies using a custom LabVIEW (software version 23.3.1f1) script. Single-molecule fluorescence spots from the raw movies were localized using custom IDL scripts (versions 8.4 and 9.1) and converted into raw fluorescence trajectories. Raw fluorescence trajectories were corrected for bleed through of the donor fluorescence into the acceptor channel. Apparent FRET efficiencies were calculated as the ratio of acceptor intensity divided by the sum of the donor and acceptor intensities. Two mechanical shutters (LS-3, Uniblitz, Vincent Associates) were placed in the excitation path for alternating laser excitation (ALEX). Frame acquisition and shutter synchronization was obtained using a home-built negative edge triggered JK flip-flop circuit (SN74LS112AN, Texas Instruments) using the “Fire” output of the EMCCD camera as the input clock. IDL scripts were modified accordingly to locate single molecules and extract fluorescence trajectories. Trajectories were only included if they met specific criteria. For the smFRET looping experiments, to be considered in the dataset, trajectories had to include a single donor bleaching step and a minimal donor intensity of 1000 AU. Furthermore, for the experiments where the red laser was turned on at the beginning of imaging for a few frames before the green laser was turned on, the trajectories had to show a clear acceptor signal to be included in the dataset. For the UL9-atto647N binding and DNA capture experiments, where alternating laser excitation was used, the trajectories had to show clear acceptor and donor signal to be included in the dataset.

### Synthesis of λ-DNA with a single biotin at each end

The 12 nt overhang ends of bacteriophage λ DNA were modified to each contain a single biotin attachment based on literature^102^. 5′-phosphorylated and 3′-biotinylated DNA oligonucleotides, LDNA_biot_end_1 (5′-pAGGTCGCCGCCC-TEG-Biotin-3′) and LDNA_biot_end_2 (5′-pGGGCGGCGACCT-TEG-Biotin-3′), were purchased from Integrated DNA Technologies. The oligonucleotides (1.2 pmol) were annealed to the λ-DNA ends sequentially (0.4 pmol, Thermo Scientific SD0011) by incubating the mixture at 70 °C for 15 min in 400 µl 1× T4 DNA buffer (NEB B0202), followed by slow-cooling to room temperature. The annealed products were then ligated by adding T4 DNA ligase (320 units, NEB M0202) and ATP (400 nmol, Thermo Scientific R0441) and incubated at room temperature for 2 h. After annealing and ligation of both oligos, the ligase was heat-inactivated by incubating the mixture at 65 °C for 20 min.

### Double-tethered DNA experiments at TIRF

All steps were performed on the home-built objective-based TIRF microscope with the outlet tubing connected to a syringe pump. Biotin-PEG-passivated home-built glass flow chambers (see sections above for more details) were incubated with 0.1 mg/ml neutravidin in T50 (50 mM NaCl and 50 mM Tris-Cl (pH 7.5)) for 5 min, then blocked for 10 min with Pluronic F-127 (0.5% w/v in T50). End-biotinylated cos6 (see C-trap section for details) or λ-DNA with a single biotin at each end was immobilized at a flow rate of ~ 5 µl/min and a total of 300 µl of ~2–4 pM DNA. Once the DNA was immobilized, slow flow rates (2–5 µl/min) were used to prevent damage to the DNA template. A second blocking step was performed using 1.7 µM biotin in UL9 buffer (50 mM NaCl, 0.1 mg/ml BSA, 40 mM Tris-Cl (pH7.5), 1 mM MgCl_2_, 0.5 mM DTT, and 50 mM potassium glutamate). Immobilized DNA was imaged in UL9 buffer with 50 nM SYTOX orange (sxo, ThermoFisher) and glucose-oxidase scavenger system (8 mg/ml D-glucose, 1 mg/ml oxidase, and 40 µg/ml catalase) in the presence or absence of 10 nM UL9-atto647N or 20 nM UL9. To image the sxo signal, the excitation laser with the wavelength of 532 nm, and to image the atto647N signal, the 637 nm wavelength laser were used. For emission, a 534/640 bandpass filter (Chroma, DC/ZET532/640 m) with a compatible dichroic mirror was used. The fluorescence was spectrally separated using a MultiSplit (Cairn Research) housing the following dichroic filters: T500lpxr UF2, T635lpxr UF2, and T725lpxr UF2 (Chroma). The separated fluorescence emission was projected onto quadrants of a sCMOS (ORCA Fusion, Hammamatsu) camera. Data was collected as raw movies using HCImage Live (version 4.6.1.3, Hammamatsu). Imaging data was analyzed using in-house Python (version 3.9.16) scripts. After co-localization of the separate green and red channels using Python, final images were merged, cropped, and colored using FIJI/ImageJ^103^ (version 2.3.0/1.53q). Kymographs were generated by selecting an area covering the DNA molecule and then tiled over time. To assess the intensity of the individual loops, the area of the spots was selected, and the overall signal intensity was normalized to the number of pixels. Plots were generated using home-built Python scripts.

### Electrophoretic mobility shift assays (EMSAs)

For the EMSAs, the same DNA substrate was used as for the UL9-atto647N TIRF-based binding assay. Cy3-labelled ssDNA AM057 and biotinylated ssDNA AM071 were annealed at a concentration of 1 µM in T50 (50 mM NaCl, 50 mM Tris-Cl, pH 7.5) by first incubating for ~45 s at 95 °C and 10–15 min cool down at room temperature. 10 nM annealed template DNA was incubated with UL9 protein in UL9 buffer (50 mM NaCl, 0.1 mg/ml BSA, 40 mM Tris-Cl (pH7.5), 1 mM MgCl_2_, 0.5 mM DTT and 50 mM potassium glutamate) at the indicated concentrations with or without 1 mM ATP or ATPγS for 5 min at room temperature. Loading buffer was added and the samples were run on a 1% agarose gel in TBE for 20 min at 100 V. Cy3 signal was imaged using an Amersham Imager 600 and the bands were quantified as described below.

### ATPase assay

UL9 ATPase activity was assessed using the EnzCheck kit (ThermoFisher, EnzCheck Phosphatase Assay Kit) following the manufacturer’s protocol and a readout at 360 nm wavelength. To stimulate ATPase activity, a final concentration of 10 µM M13 DNA (ssDNA, circular, NEB) was added. Assay conditions: 200 nM UL9 (or UL9 E175A), 50 mM NaCl, 5 mM MgCl_2_, 2 mM ATP, 100 µg/ml BSA, 50 mM Tris (pH 8), measured at room temperature.

### Microscale thermophoresis ATP binding assay

Microscale thermophoresis (MST)^104^ was used to determine ATP binding to either wildtype (wt) or mutant (E175A) UL9. The interaction between UL9 (wt) and UL9 (E175A) with ATP was measured on a temperature equilibrated Monolith^TM^ NT.115 (NanoTemper), using a fluorescent ATP labelled with AlexaFluor 647 (Thermo Fisher). For each set of measurements, a twofold serial-dilution of UL9 (wt) or UL9 (E175A) was prepared at 2× final concentration (starting at 2 μM) in an MST-Buffer [100 mM Tris-HCl pH 7.5, 150 mM NaCl, 1 mM TCEP, 1 mM MgCl_2_, 10% (v/v) glycerol, 0.005% Tween 20 and 0.1 mg/ml BSA] before mixing with an equal volume of ATP stock solution (also in an MST buffer), to yield a final concentration of 20 nM. Samples were then loaded into a Monolith^TM^ NT.115 MST Premium Coated capillaries (NanoTemper). LED Power was set to 40%, and MST power to 60% for all experiments. All other settings were left at default values. Data plotting and fitting were performed using IgorPro8 (version 8.04, WaveMetrics) using the temperature jump data.

### Data analysis

#### Analysis of smFRET traces for the oriS-looping assays

To generate histograms assessing the different FRET states, the sections of the traces showing FRET (or entire traces for the controls) were selected and converted into FRET efficiency traces (FRET = I_acc_/(I_acc_ + I_don_)) using MATLAB (version R2022b 9.13.0, MathWorks). Histograms were generated and fitted using IgorPro8 (version 8.04, WaveMetrics). Data displayed in histograms was normalized by the sum of all datapoints within one condition to obtain probability.

#### Kinetic analysis of UL9-atto647N binding and DNA capture assays

Raw atto647N/Cy5 traces were first converted into a format that could be used by tMAVEN using an in-house Matlab script (MATLAB version R2022b 9.13.0). We then used Python (versions 3.11.11 and 3.9.16) to convert the file into an .h5 file. In tMAVEN^105^ (version 0.2.0), we first performed a global Hidden-Markov Modelling (HMM) for all traces of each experiment (vbConsensus), assuming two states and using the default parameters. Since we only assessed binding/no binding, we didn’t distinguish any multimer binding states. After HMM modelling, we removed the traces, which were over- or under-fitted. Dwell times from the idealized FRET trajectories were extracted using custom MATLAB (version R2022b 9.13.0, MathWorks) scripts. The data was then plotted as histograms with IgorPro8 (version 8.04, WaveMetrics) and fit to a single or double exponential distribution to determine *k* and *τ* (1/*k*).

Single exponential:

1 $$f\left(t\right)=A{e}^{-{kt}}$$

Double exponential:

2 $$f\left(t\right)={A}_{1}{e}^{-{k}_{1}t}+\,{A}_{2}{e}^{-{k}_{2}t}$$

Standard deviation of τ was determined as follows:

3 $$\Delta \tau=\frac{\Delta k}{{k}^{2}}$$

Average lifetime (*τ*_avg_) for UL9-atto647N binding or DNA capture was calculated from the double exponential fit as follows:

4 $${\tau }_{{avg}}=\frac{{A}_{1\,}\,{\tau }_{1}^{2}\,{+\,A}_{2\,}\,{\tau }_{2}^{2}}{{A}_{1\,}\,{\tau }_{1}\,{+\,A}_{2\,}\,{\tau }_{2}}$$

The apparent dissociation constant of UL9-atto647N binding to oriS was calculated as follows:

5 $${K}_{d}=\frac{{\tau }_{{off}\,{avg}}}{{\tau }_{{on}\,{avg}}}$$

The error of *K*_*d*_ was calculated as follows^106^:

6 $$\Delta {K}_{d}=\,\frac{{\tau }_{{off\; avg}}}{{\tau }_{{on\; avg}}}\sqrt{{\left(\frac{{\Delta \tau }_{{off\; avg}}}{{\tau }_{{off\; avg}}}\right)}^{2}+{\left(\frac{{\Delta \tau }_{{on\; avg}}}{{\tau }_{{on\; avg}}}\right)}^{2}}$$

#### Calculation of loop sizes from C-trap force-extension curves (FECs)

FEC data was extracted from.h5 files (acquired with Lumicks Bluelake) using a home-built Python (3.11.11 and 3.9.16) script^66^. Loop sizes were then assessed in MATLAB (version R2022b 9.13.0, MathWorks) using a home-built script. The PeakFinder function was used to first identify the coordinates (DNA length and rupture force) of the rips and then the corresponding point at the same force (DNA length after loop rupture). The difference between the initial length at the rupture point and the DNA length after the rupture was determined and converted into an approximate number of base pairs with 1 bp = 0.338 nm^107^. Only datapoints showing correctly identified peaks were included. Plots were generated with IgorPro8 (version 8.04, WaveMetrics). Histograms showing the distribution of loop sizes in Fig. 3 were generated with Python (3.9.16). The data was fit to a gamma distribution:

7 $$f(x)=\frac{{x}^{n-1}{e}^{-x/{l}_{0}}}{\Gamma ({{{\rm{n}}}})\,{l}_{0}^{n}}$$

with *x* being the loop length, *n* the shape *l*_*0*_ the scale factor.

#### Determination of UL9-atto647N binding site from C-trap kymographs

Trace length and location from kymographs of UL9-atto647N binding to the HSV-1-derived cos6 template were analyzed using a home-built Python (versions 3.11.11 and 3.9.16) script. Distance from the center of the template in each frame from the entire trace was then calculated in MATLAB (version R2022b 9.13.0, MathWorks) and histograms were plotted using IgorPro8 (version 8.04, WaveMetrics). Kymographs showing binding of aggregates were excluded from the analysis.

#### Analysis of electrophoretic mobility shift assays (EMSAs)

The fraction of bound (shifted) DNA for each lane was determined using the Bio-Rad Laboratories Inc. Image Lab Software (Version 6.1). Percentage of bound DNA was plotted against UL9 concentration of 3 independent experiments and the Hill equation was fitted globally using a maximum likelihood estimation (MLE) in Python (version 3.9.16).

Fitted Hill equation^108,109^ with *K*_1/2_ for the concentration where half of the substrate is bound, *c* for UL9 concentration, *n* for Hill coefficient, and *V*_max_ for maximum bound fraction (*V*_0_ = 0):

8 $$f(c)=\frac{{V}_{max }{c}^{n}}{{K}_{1/2}^{n}+\,{c}^{n}}$$

To determine *K*_*d*_ we used *K*_*d*_ = *K*_1/2_^n^ and to calculate the error we used^106^:

9 $${\left(\frac{\Delta {K}_{d}}{{K}_{d}}\right)}^{2}={\left(\frac{n\Delta {K}_{1/2}}{{K}_{1/2}}\right)}^{2}+{\left({{{\mathrm{ln}}}}\left({K}_{1/2}\right)\Delta n\right)}^{2}$$

### Reporting summary

Further information on research design is available in the Nature Portfolio Reporting Summary linked to this article.

## Supplementary information

Supplementary Information

Description of Additional Supplementary Files

Supplementary Movie 1

Reporting Summary

Transparent Peer Review file

## Source data

Source Data

## Data Availability

The smFRET, optical tweezer, and TIRF datasets generated during the current study as well as the Source Data file including uncropped EMSA and Coomassie gels, are available on Zenodo (10.5281/zenodo.20011157)^110^ and from the corresponding authors upon request. Materials are available upon request with completion of a standard Materials Transfer Agreement. Source data are provided with this paper.

## Code availability

Code for single-molecule data analysis is available on GitHub (https://github.com/singlemoleculegroup).
