# Supplementary material for: HSV-1 origin binding protein UL9 forms intermolecular DNA tethers and intramolecular DNA loops
Source: Nat Commun. 2026 Jun 29;17:8110. doi: 10.1038/s41467-026-74361-w (PMC13458718; doi:10.1038/s41467-026-74361-w)
Supplement: Supplementary file 1 [file 41467_2026_74361_MOESM1_ESM.pdf]

Supplementary Information

HSV-1 origin binding protein UL9 forms intermolecular DNA tethers and intramolecular DNA loops

Anita F. Meier[1,2]*, Jan Vuckovic[1,2], Paul Girvan[1,2], Adam S. B. Jalal[2], Erin Cutts[2,3], Theodora Brophy[1,2], Benjamin Ambrose[1,2] and David S. Rueda[1,2]*

[1] Section of Virology, Dept. Infectious Disease, Faculty of Medicine, Imperial College London, Du Cane Road, London W12 0HS, UK
[2] Single-Molecule Biophysics Group, MRC Laboratory of Medical Sciences, Du Cane Road, London W12 0HS, UK
[3] Current address: School of Biosciences, The University of Sheffield, Sheffield, S10 2TN, UK

* Correspondence to: anita.meier@imperial.ac.uk; david.rueda@imperial.ac.uk

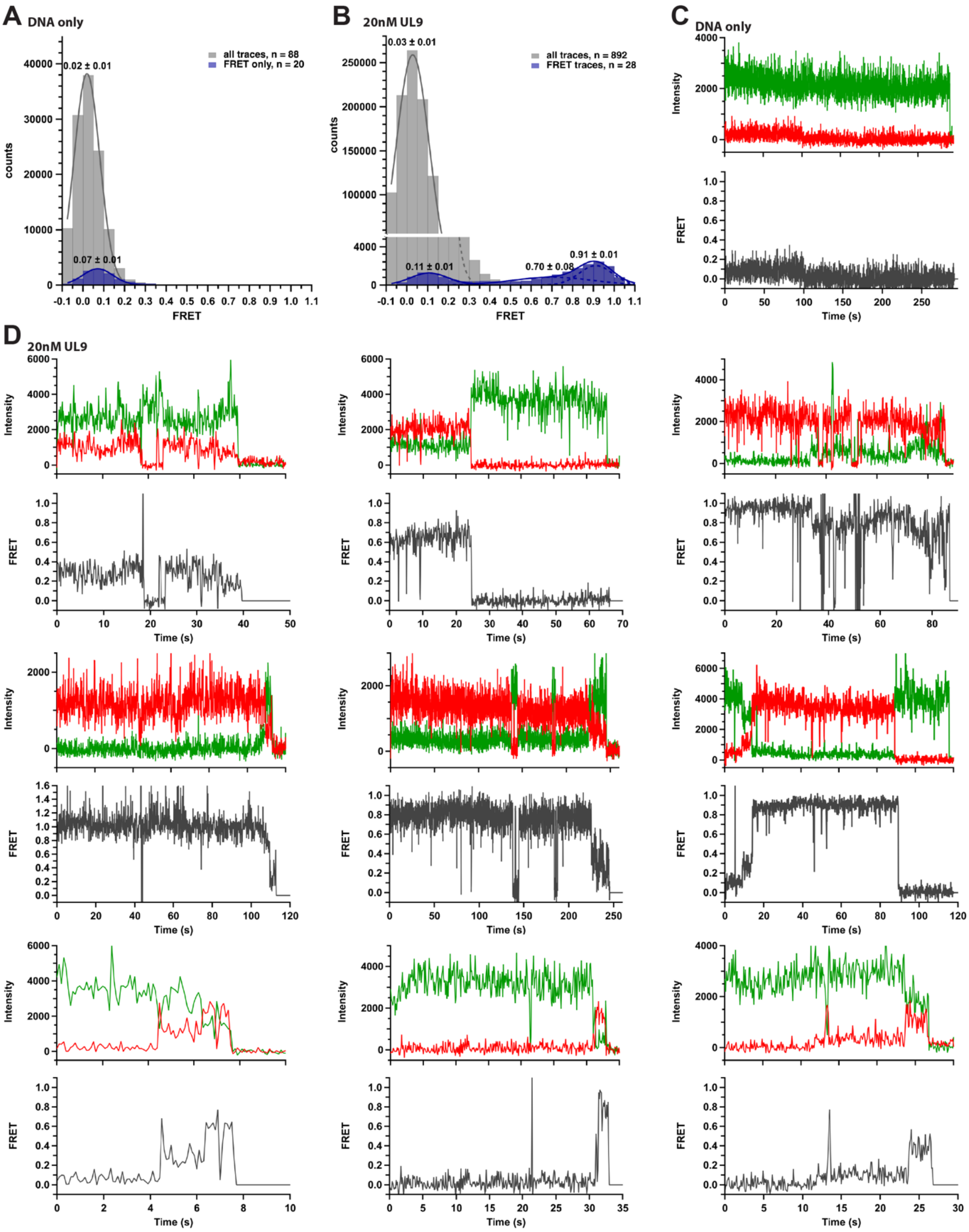

Supplementary Figure 1: Additional data of the smFRET oriS-looping assay. Histogram of FRET efficiency including all traces (grey) or only traces showing FRET (blue) in the absence (DNA only, **A**) or presence of 20 nM UL9 (**B**). The data was fit to Gaussian distributions (dashed lines) with the center of each distribution and the overall fit (solid line) indicated. n = number of traces. Data was collected from a minimum of 3 independent experiments. Example traces of Cy3- (donor) and Cy5 (acceptor)-labelled oriS DNA under acceptor excitation in buffer (**C**) or in the presence of 20 nM UL9 (**D**) with donor (green) and acceptor (red) signal in the panel in the top row and the FRET efficiency (grey) in the bottom row. Source data are provided as a Source Data file.

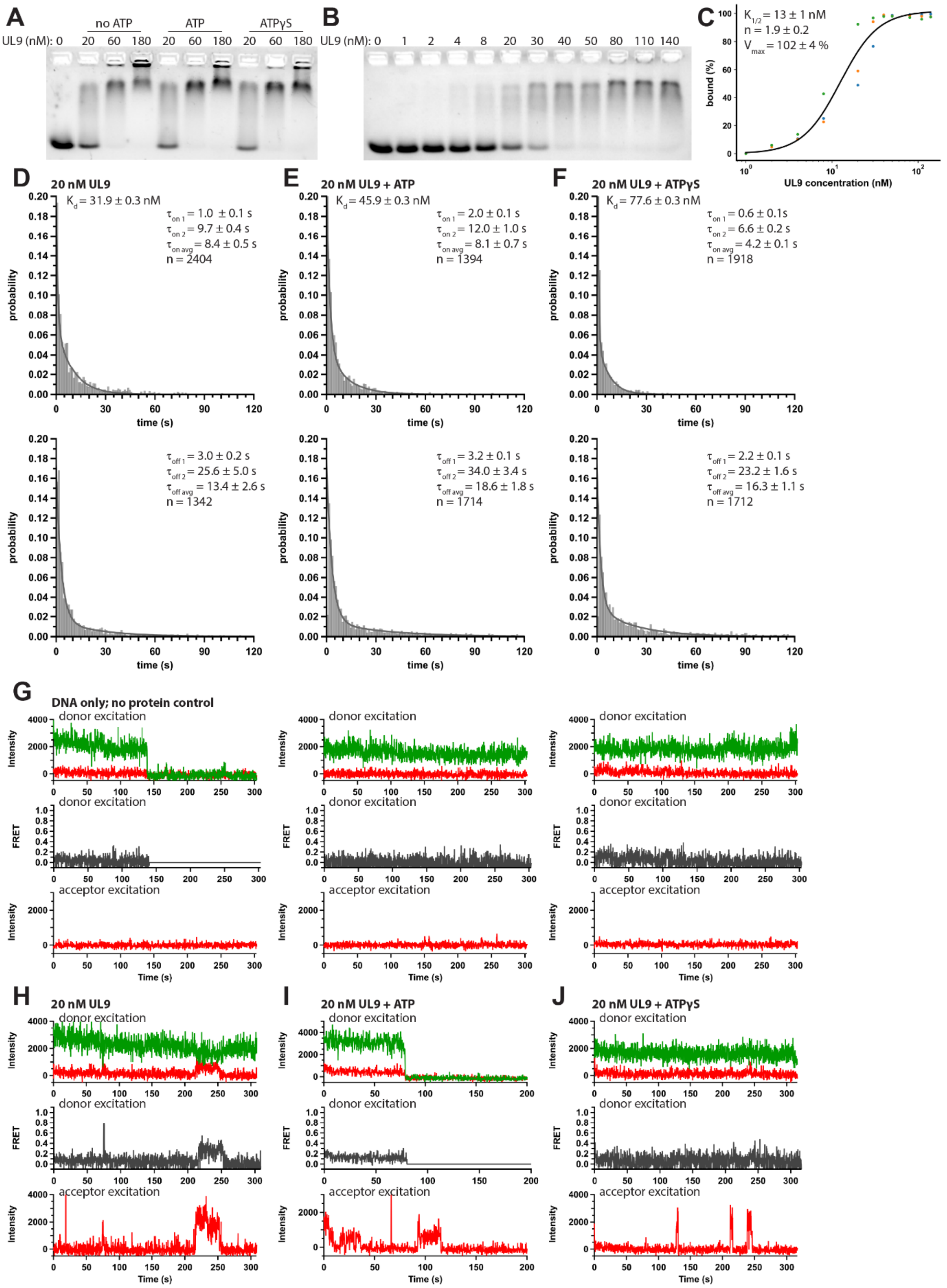

Supplementary Figure 2: Assessment of UL9 binding to the oriS template. Representative electrophoretic mobility shift assay (EMSA) images showing UL9 binding to Cy3-oriS DNA in the absence or presence of 1 mM ATP or ATPγS (**A**) and in the absence of ATP (**B**) with UL9 concentrations indicated. **C** Quantification of shifted fraction of template DNA from 3 independent EMSA gels, colors indicate separate experiments (blue, green and

orange). Global Hill fit (f(conc.) = (V_{max} conc.^{n})/($K_{1/2}^n$ + conc.^{n})) across replicates is shown. Since $K_{1/2}^n$ equals K_d, we obtained an apparent K_d of 131 ± 70 nM. Uncropped gel images are included in the Source Data file. **D – F** Dwell time determination of UL9-atto647N on immobilized Cy3-oriS template DNA**.** Histograms of UL9-atto647N dwell times in the bound (top row) and unbound (bottom row) state in the absence (**D**) and presence of 1 mM ATP (**E**) or 1 mM ATPγS (**F**). n = number of transitions. Data was collected from 3 independent experiments per condition. **G – J** Example traces of UL9-atto647N binding to immobilized Cy3-oriS DNA using alternating laser excitation (ALEX). Donor (green) and acceptor (red) signal under donor excitation is shown in the top row, FRET signal (grey) under donor excitation is shown in the middle row and acceptor signal (red) under acceptor excitation is shown in the bottom row. **G** DNA only control traces. Example traces in the presence of 20 nM UL9-atto647N only (**H**) or in the presence of 1 mM ATP (**I**) or ATPγS (**J**). Source data are provided as a Source Data file.

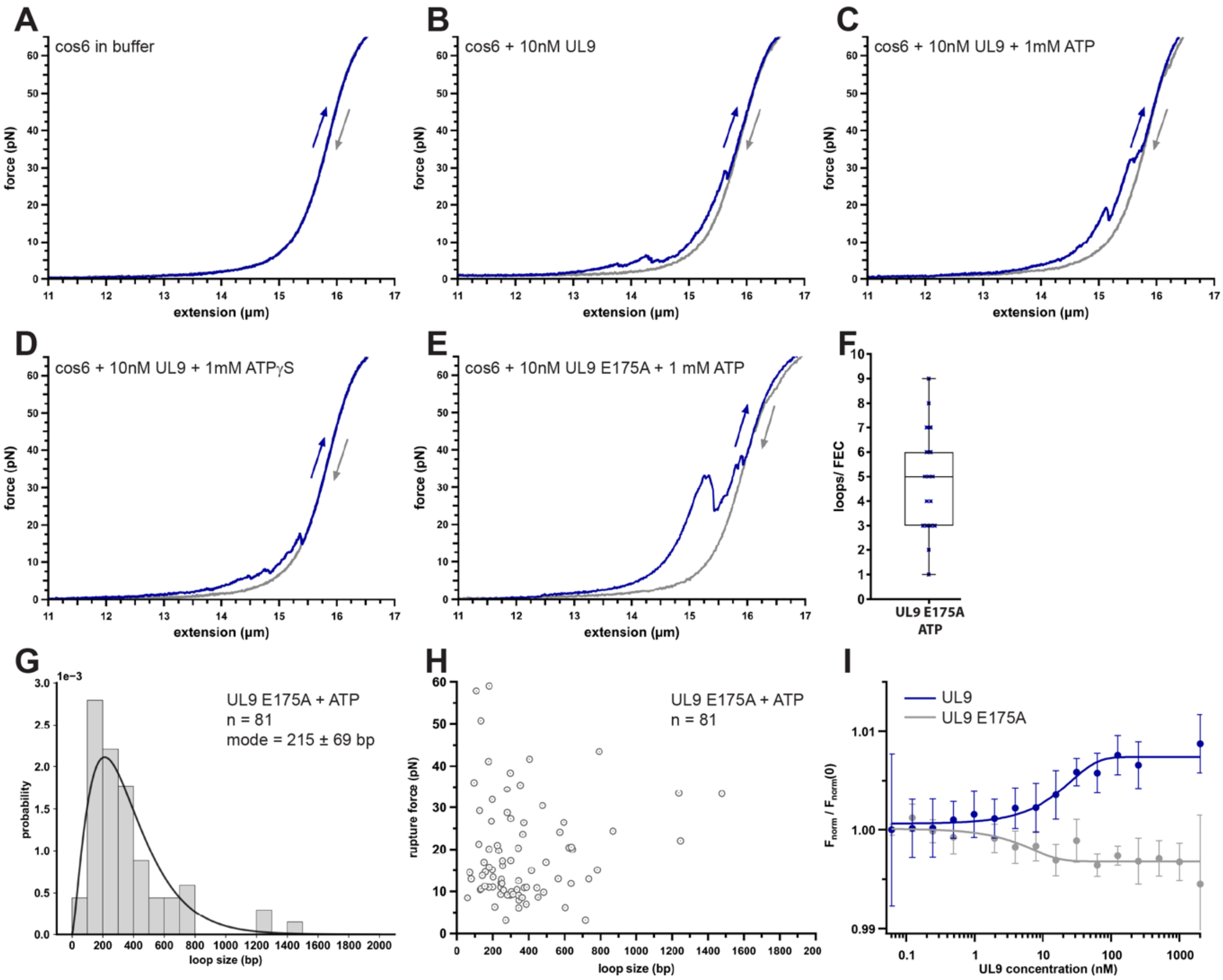

Supplementary Figure 3: Additional data showing UL9-induced DNA loop formation and ATP binding to UL9. A-E Force-extension curves (FECs) of cos6 in buffer only (**A**) and in the presence of 10 nM UL9 alone (**B**) or with 1mM ATP (**C**)/ ATPγS (**D**) or 10 nM UL9 E175A (**E**) with 1 mM ATP as indicated. **F** Boxplot showing number of loops per cos6 DNA molecule in the presence of UL9 E175A and ATP (4.8 ± 2.2 SD, median = 5, Q1 = 3, Q3 = 6, min =1, max = 9, n = 17), n = number of FECs. **G** Histogram including a gamma fit of the loop size distribution in the presence of 10 nM UL9 E175A and 1 mM ATP, n = number of loops. **H** Scatter plot showing the correlation of loop size versus rupture force on cos6 DNA in the presence of 10 nM UL9 E175A and 1 mM ATP, n = number of loops. **I** MST data showing ATP binding to wildtype (wt, blue) as an increase in $F_{norm}/F_{norm}(0)$ with UL9 concentration, and absence of ATP binding to the E175A mutant ($F_{norm}/F_{norm}(0)$ decrease, grey). Averages of 3 measurements each are shown including standard deviation and sigmoid fit. The experiment was performed twice. Source data are provided as a Source Data file.

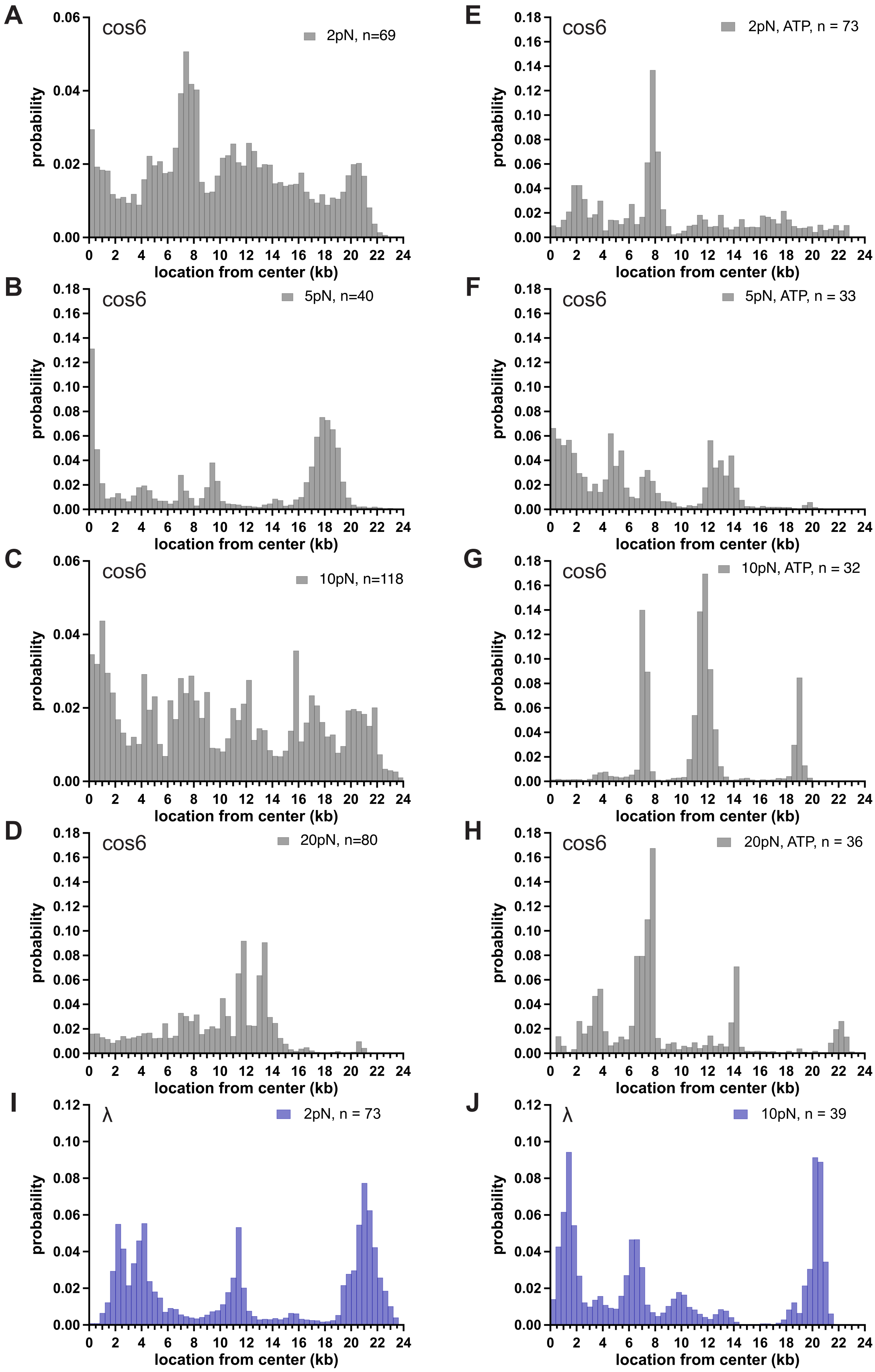

Supplementary Figure 4: Additional data of UL9-atto647N binding locations at different forces and templates. Histograms of UL9-atto647N (10 nM) binding location on cos6 (grey, **A – H**) or λ (blue, **I and J**) DNA plotted from the center of the template held at 2 to 20 pN in

the absence or presence of 1 mM ATP as indicated, n = number of UL9-atto647N traces. Data was collected from at least 3 independent experiments per condition. Source data are provided as a Source Data file.

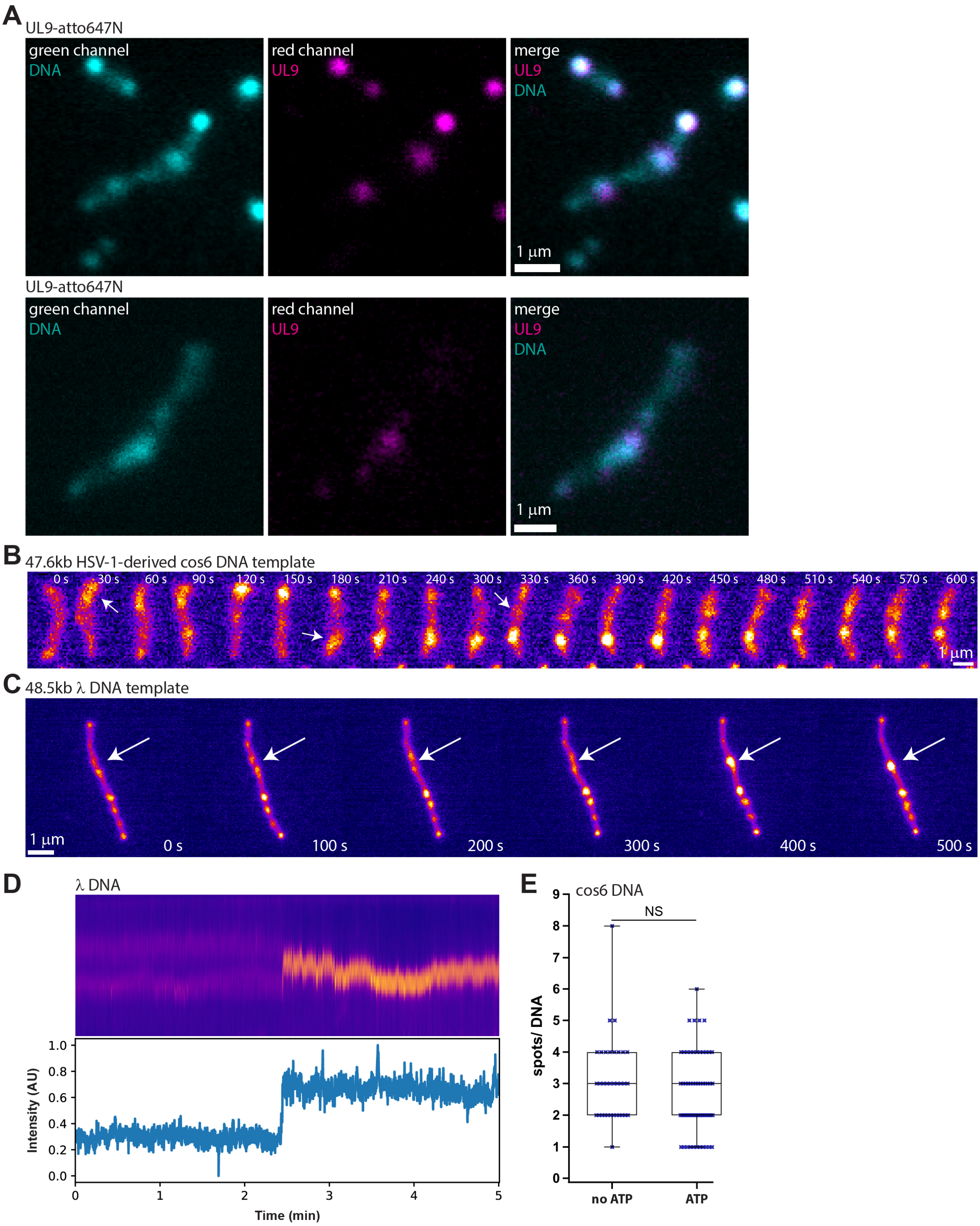

Supplementary Figure 5: UL9 induced loops on double tethered DNA. A TIRF microscopy images of double-tethered cos6 DNA (turquoise) in the presence of 10 nM UL9-atto647N (purple). Data with atto647N-labelled UL9 was collected in one experiment. Microscopy images at indicated timepoints of an emerging loop (arrow) on double-tethered cos6 (**B**) in the presence of 4 nM UL9 and 1 mM ATP or λ DNA (**C**) in the presence of 10 nM UL9 with arrows pointing to the sites of newly emerging loops. Scale bar indicates 1 µm. 3 independent experiments with λ DNA and unlabeled UL9 and 6 independent experiments with cos6 DNA and unlabeled UL9 were performed. **D** Kymograph of a zoomed-in region of the emerging loop with the pixel intensity below. Microscopy images in B – D are shown using the fire LUT ranging

from the lowest (black) to the highest (white) fluorescence intensity level. **E** Boxplot showing number of loops per cos6 DNA template in the presence of UL9 without (2.7 ± 1.3 SD, median = 3, Q1 = 2, Q3 = 4, min = 1, max = 8, n = 59) or with 1 mM ATP (3.1 ± 1.3, median = 3, Q1 = 2, Q3 = 4, min = 1, max = 6, n = 34, p = 0.16). Data was collected from two independent experiments per condition. Blue markers indicate loop counts from individual DNA molecules. Two sample t-test was performed with NS for $p \geq 0.05$ ($p = 0.157$). Source data are provided as a Source Data file.

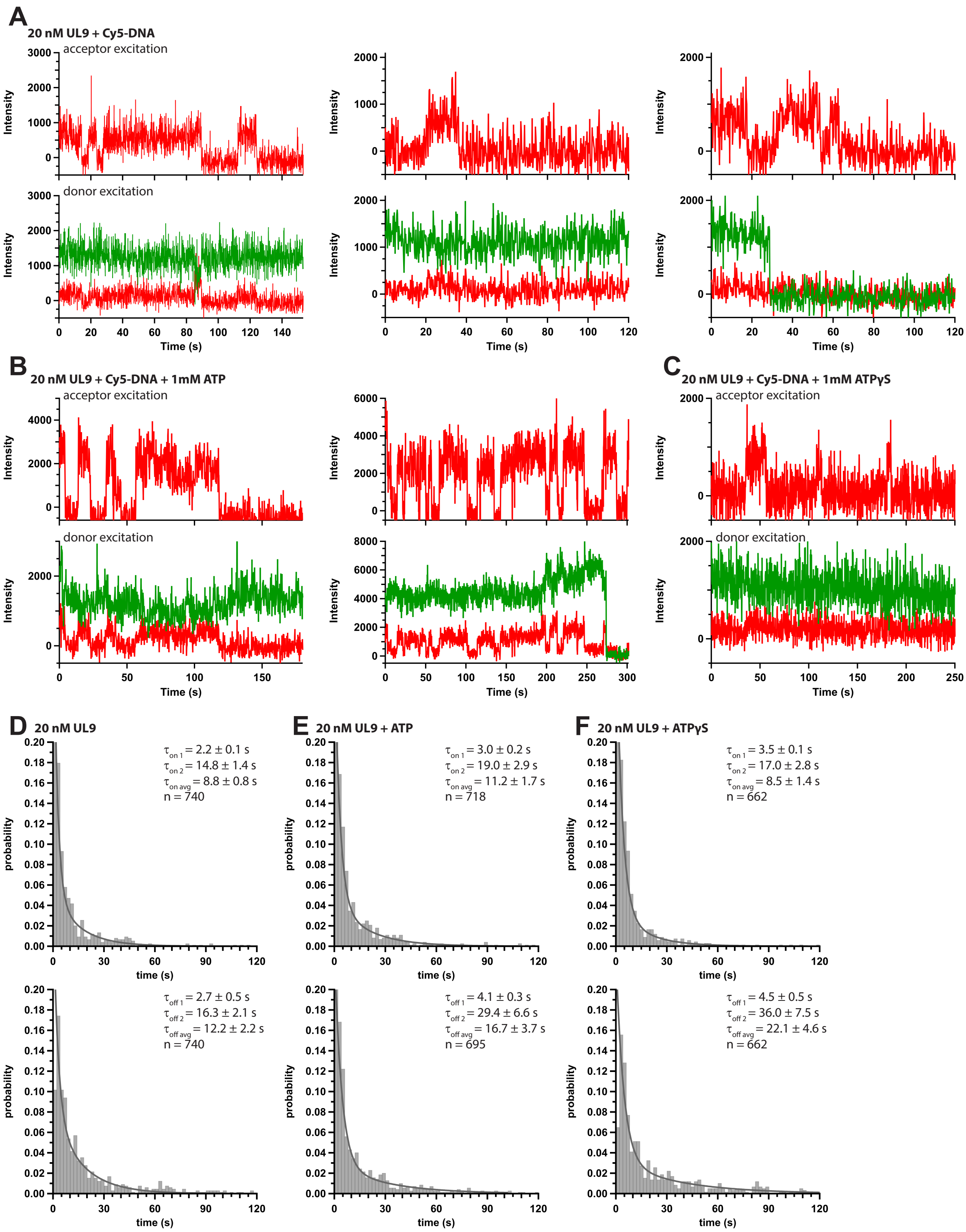

Supplementary Figure 6: UL9 captures DNA in trans. Additional smFRET traces of immobilized Cy3-labelled DNA (green) in the presence of 20 nM UL9, 10 nM Cy5-DNA (red) in the absence (**A**) or presence of 1 mM ATP (**B**) or 1 mM ATPγS (**C**) under acceptor (top) or donor (bottom) excitation. DNA Dwell time analysis of Cy5-DNA in the absence (**D**) or presence of 1 mM ATP (**E**) or 1 mM ATPγS (**F**). n = number of state transitions. Data was collected from 3 (UL9), 2 (ATPγS) and 1 (ATP) independent experiments per condition. Source data are provided as a Source Data file.

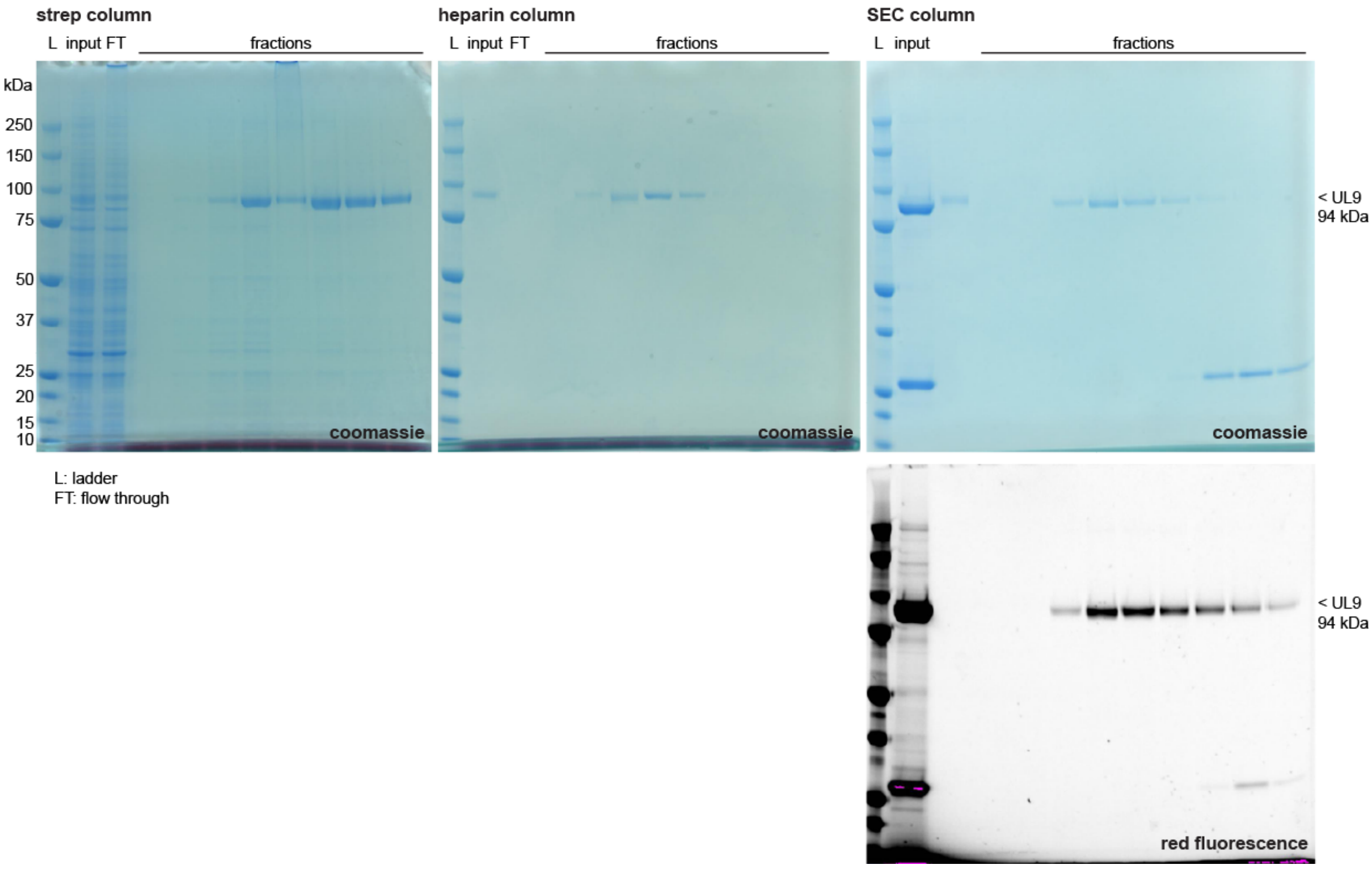

Supplementary Figure 7: UL9 protein purification. Coomassie blue stained SDS PAGE gels of fractions collected during UL9 purification. One gel per column (strep/ heparin and SEC) is shown. L = ladder, input = column input fraction, FT = unbound flow-through. UL9 has an expected mass of 94 kDa. UL9 was labelled with atto647N after the heparin and before the SEC column. The SEC-column gel was also imaged for red fluorescent signal. See methods for further details.

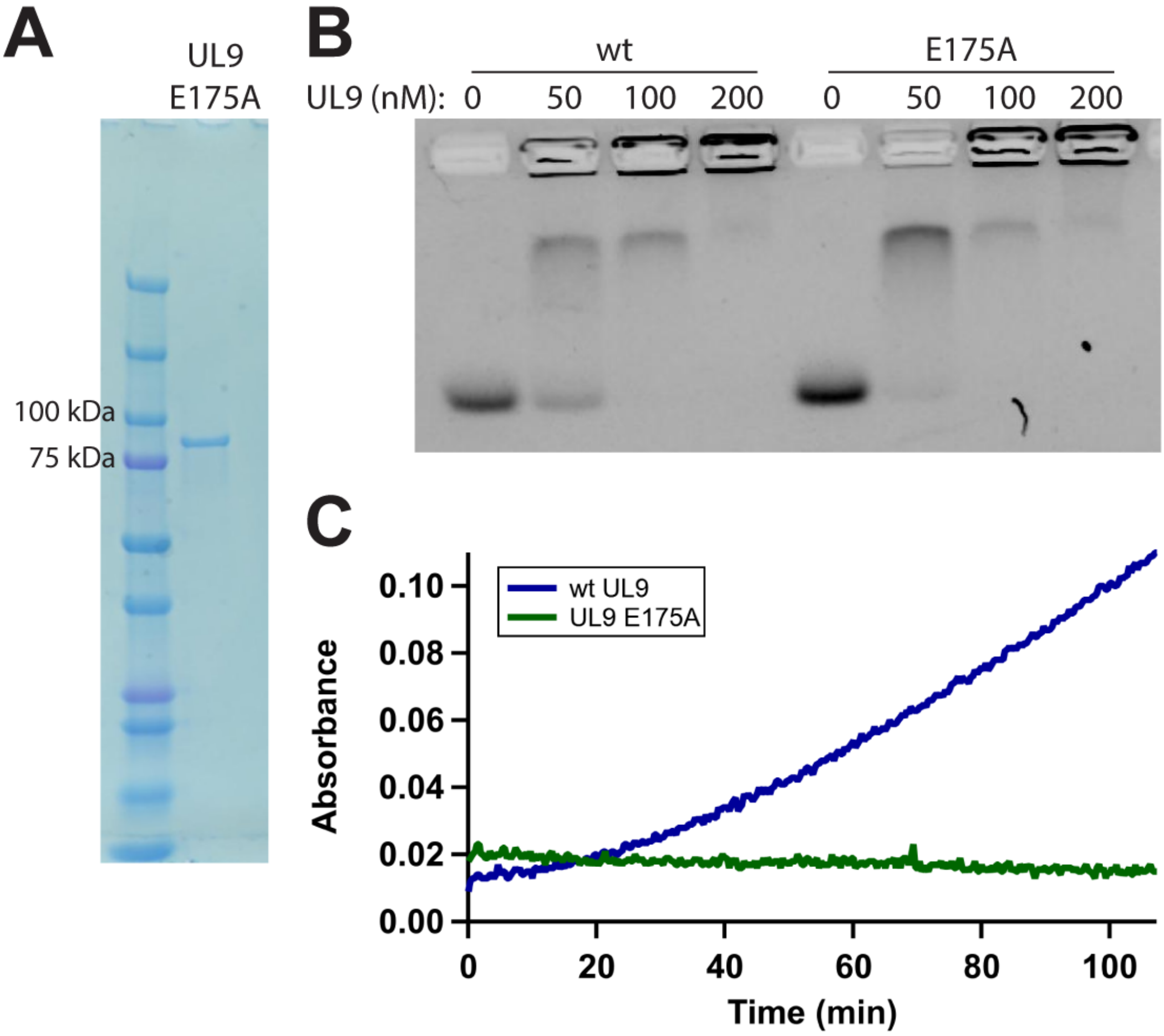

Supplementary Figure 8: Characterization of UL9 E175A. A Coomassie stained SDS PAGE gel showing purified UL9 E175A (expected size: 94 kDa). **B** Electrophoretic mobility shift assay (EMSA) image showing wildtype (wt) and mutant (E175A) UL9 binding to Cy3-oriS DNA. This experiment was performed once. **C** ATPase assay of wt (blue) and mutant (E175A; green) UL9. This experiment was performed once. Source data are provided as a Source Data file.
