# Supplementary material for: HSV-1 origin binding protein UL9 forms intermolecular DNA tethers and intramolecular DNA loops
Source: Nat Commun. 2026 Jun 29;17:8110. doi: 10.1038/s41467-026-74361-w (PMC13458718; doi:10.1038/s41467-026-74361-w)
Supplement: Supplementary file 2 [file 41467_2026_74361_MOESM2_ESM.pdf]

Description of Additional Supplementary Files

File name: **Supplementary Movie 1**

Description: **UL9 induces loops into double-tethered cos6 DNA.** Movies of a double-tethered cos6 DNA in the absence or presence of 4 nM UL9. Frame rate during recording: 100 ms, timestamp indicates duration (in min:sec) during original recording. Images were colored using the fire LUT to emphasize differences in intensity spanning the lowest (black) and highest (white) intensity value.
