# Supplementary material for: HSV-1 origin binding protein UL9 forms intermolecular DNA tethers and intramolecular DNA loops
Source: Nat Commun. 2026 Jun 29;17:8110. doi: 10.1038/s41467-026-74361-w (PMC13458718; doi:10.1038/s41467-026-74361-w)
Supplement: Supplementary file 3 [file 41467_2026_74361_MOESM4_ESM.pdf]

nature portfolio

Corresponding author(s): Anita Meier and David Rueda

Last updated by author(s): May 9, 2026

Reporting Summary

Nature Portfolio wishes to improve the reproducibility of the work that we publish. This form provides structure for consistency and transparency in reporting. For further information on Nature Portfolio policies, see our Editorial Policies and the Editorial Policy Checklist.

Statistics

For all statistical analyses, confirm that the following items are present in the figure legend, table legend, main text, or Methods section.

n/a	Confirmed	
☐	☒	The exact sample size (*n*) for each experimental group/condition, given as a discrete number and unit of measurement
☐	☒	A statement on whether measurements were taken from distinct samples or whether the same sample was measured repeatedly
☐	☒	The statistical test(s) used AND whether they are one- or two-sided<br>*Only common tests should be described solely by name; describe more complex techniques in the Methods section.*
☐	☒	A description of all covariates tested
☐	☒	A description of any assumptions or corrections, such as tests of normality and adjustment for multiple comparisons
☐	☒	A full description of the statistical parameters including central tendency (e.g. means) or other basic estimates (e.g. regression coefficient) AND variation (e.g. standard deviation) or associated estimates of uncertainty (e.g. confidence intervals)
☐	☒	For null hypothesis testing, the test statistic (e.g. *F*, *t*, *r*) with confidence intervals, effect sizes, degrees of freedom and *P* value noted<br>*Give P values as exact values whenever suitable.*
☒	☐	For Bayesian analysis, information on the choice of priors and Markov chain Monte Carlo settings
☒	☐	For hierarchical and complex designs, identification of the appropriate level for tests and full reporting of outcomes
☒	☐	Estimates of effect sizes (e.g. Cohen's *d*, Pearson's *r*), indicating how they were calculated

Our web collection on statistics for biologists contains articles on many of the points above.

Software and code

Policy information about availability of computer code

Data collection	Single-molecule TIRF videos were acquired using a home-built LabVIEW script (software version 23.3.1f1) or HCImage 4.6.1.3 (Hammamatsu). c-trap data was acquired using Lumicks Bluelake.
Data analysis	Single-molecule TIRF data was processed using custom made scripts in IDL (versions 8.4 and 9.1) or python (3.11.11 and 3.9.16), traces were further analyzed using in house scripts in MATLAB (R2022b 9.13.0) and tMAVEN (version 0.2.0). Double-tethered DNA imaged at the TIRF microscope was visualized using FIJI (2.3.0/1.53q) c-trap data was analyzed using home-built python (3.9.16) and MATLAB (R2022b 9.13.0) scripts. Statistical analysis was performed using IgorPro8 (8.04) and in-house MATLAB scripts. Data plots (e.g. histograms) were generated using IgorPro8 (8.04). Code for single-molecule data analysis is freely available on the group github page (https://github.com/singlemoleculegroup).

For manuscripts utilizing custom algorithms or software that are central to the research but not yet described in published literature, software must be made available to editors and reviewers. We strongly encourage code deposition in a community repository (e.g. GitHub). See the Nature Portfolio guidelines for submitting code & software for further information.

Data

Policy information about availability of data

All manuscripts must include a data availability statement. This statement should provide the following information, where applicable:

- Accession codes, unique identifiers, or web links for publicly available datasets
- A description of any restrictions on data availability
- For clinical datasets or third party data, please ensure that the statement adheres to our policy

The datasets generated and analyzed during the current study are freely available on zenodo117 (10.5281/zenodo.20011158) and from the corresponding authors on request.

Research involving human participants, their data, or biological material

Policy information about studies with human participants or human data. See also policy information about sex, gender (identity/presentation), and sexual orientation and race, ethnicity and racism.

Reporting on sex and gender	N/A
Reporting on race, ethnicity, or other socially relevant groupings	N/A
Population characteristics	N/A
Recruitment	N/A
Ethics oversight	N/A

Note that full information on the approval of the study protocol must also be provided in the manuscript.

Field-specific reporting

Please select the one below that is the best fit for your research. If you are not sure, read the appropriate sections before making your selection.

☒ Life sciences ☐ Behavioural & social sciences ☐ Ecological, evolutionary & environmental sciences

For a reference copy of the document with all sections, see nature.com/documents/nr-reporting-summary-flat.pdf

Life sciences study design

All studies must disclose on these points even when the disclosure is negative.

Sample size	No sample size calculations were performed before the experiments. Sample size for smFRET and optical tweezer data are based on data acquired from at least 3 independent repeats.
Data exclusions	Inclusion criteria for single molecule imaging data are detailed in the methods section.
Replication	Replication statements for each experiment are included in the manuscript. Sample sizes for the individual experiments are indicated in the manuscript. Exclusion criteria for smFRET/ TIRF traces are indicated in the manuscript. Replication attempts were successful.
Randomization	The experiments outlined in this manuscript are in vitro assays and therefore randomization is not relevant for this study.
Blinding	Blinding was not relevant for the experiments in this study.

Reporting for specific materials, systems and methods

We require information from authors about some types of materials, experimental systems and methods used in many studies. Here, indicate whether each material, system or method listed is relevant to your study. If you are not sure if a list item applies to your research, read the appropriate section before selecting a response.

Materials & experimental systems

n/a	Involved in the study
☒	☐ Antibodies
☐	☒ Eukaryotic cell lines
☒	☐ Palaeontology and archaeology
☒	☐ Animals and other organisms
☒	☐ Clinical data
☒	☐ Dual use research of concern
☒	☐ Plants

Methods

n/a	Involved in the study
☒	☐ ChIP-seq
☒	☐ Flow cytometry
☒	☐ MRI-based neuroimaging

Eukaryotic cell lines

Policy information about cell lines and Sex and Gender in Research

Cell line source(s)	Spodoptera frugiperda Sf9, Thermo Fisher Scientific, 11496015<br>Trichoplusia ni High Five,Thermo Fisher Scientific, B85502
Authentication	Since the cell lines were bought from a company, they were not authenticated.
Mycoplasma contamination	Cell lines were not tested for mycoplasma.
Commonly misidentified lines<br>(See ICLAC register)	None used.

Plants

Seed stocks	N/A
Novel plant genotypes	N/A
Authentication	N/A
