# Supplementary material for: HSV-1 origin binding protein UL9 forms intermolecular DNA tethers and intramolecular DNA loops
Source: Nat Commun. 2026 Jun 29;17:8110. doi: 10.1038/s41467-026-74361-w (PMC13458718; doi:10.1038/s41467-026-74361-w)
Supplement: Supplementary file 4 [file 41467_2026_74361_MOESM5_ESM.pdf]

nature portfolio

Peer Review File

HSV-1 origin binding protein UL9 forms intermolecular DNA tethers and intramolecular DNA loops

Corresponding Author: Professor David Rueda

This file contains all reviewer reports in order by version, followed by all author rebuttals in order by version.

Version 0:

Reviewer comments:

Reviewer #1

(Remarks to the Author)
Review for "HSV-1 origin binding protein UL9 forms intermolecular DNA tethers and intramolecular DNA loops"

In "HSV-1 origin binding protein UL9 forms intermolecular DNA tethers and intramolecular DNA loops", Meier and colleagues have investigated how UL9, a replication origin binding protein, associates with the two nearby origin sites and whether, upon binding, the DNA-associated UL9s form a loop. To this end, they use several single-molecule technologies (optical tweezers with fluorescence, TIRF-based FRET, flow stretching) to determine the mode of binding of UL9, its specificity and its ability to form DNA loops. Overall, the manuscript is well-structured with well-designed experiments that adequately answer the questions of the authors. The topic is new and important, given the prevalence of herpes virus, this new study will pave the way to future important work. I would therefore highly recommend it for publication.

I have some minor points that I would appreciate the authors fix before publication:

1- Figure 1D: The trace starts at high FRET and DNA looping by UL9 has equilibrated: can we have data where we observe this process dynamically, i.e. trace starting before adding UL9 and ending with a bent DNA in the presence of UL9? (the bottom right corner trace of Fig. S1 is a good example, though a single one).

2- l. 158-164: the whole paragraph on how the gamma distribution scale parameter (Fig. 3F-H) describe the role of ATP needs to be better written and explain for the broad readership of Nature Communications. I am not familiar with the model of ref. 42, and I should not have to read this reference to understand how the authors use the model to reach their conclusion. Please describe rapidly the model and how you apply it. For example, I don't understand why an increase in the gamma distribution exponent says something about ATP binding and DNA looping. Also, the sentences in the paragraph are rather long.

3- In Figure 4, the authors show that UL9 binds more specifically at oriS at low force than at high force. Don't you think this result argue in favor of DNA bending by UL9 at the origin?

Minor comments:

4- In the abstract:

"the HSV-1 origin binding protein UL9 binds two closely located binding sites" a lot of binding here.

5- l.66-67: "Despite being a superfamily II helicase, UL9 requires a 3' single-stranded DNA (ssDNA) overhang to mediate strand separation" Why despite being a SF2 helicase is in opposition to needing a 3' single-stranded DNA?

6- l.76-77 "and access to the ssDNA to other replication factors and replisome assembly" I would rather re-write this as: "and enable other replication factors to access the ssDNA and subsequent replisome assembly"

7- l.79: "which eliminate ensemble averaging": in your case, more importantly, the ability to manipulate and observe the DNA

molecule and capture such looping is the most important, isn't it?

8- l.217-218: you use the verb "tethers": I think binds or attaches is more adapted in this context. Same l.471.

(Remarks on code availability)

Reviewer #2

(Remarks to the Author)
This manuscript entitled "HSV-1 origin binding protein UL9 forms intermolecular DNA tethers and intramolecular DNA loops" describes a novel previously unrecognized behavior for the HSV origin binding protein, UL9. The authors present data suggesting that UL9 can tether two OriS DNA molecules intermolecularly, forming large DNA loops. This surprising result is of considerable interest as it is possible that intermolecular interactions between separate genomes could lead to recombination events that could alter models for HSV DNA replication. The actual data presented are for the most part of high quality and support interpretation that intermolecular interactions can occur. However, the background material presented is incomplete and relies heavily on a outdated model suggesting that the HSV genome circularizes during replication and replicates by rolling circle replication. Considerable evidence has been published over the last 20 years that indicate that HSV DNA replication is much more complex. The manuscript as written contains misleading and incompletely referenced statements. Thus, although the major finding of this paper of long-distance intermolecular looping is extremely interesting, major revision as well as additional experiments will need to be addressed before this manuscript is suitable for publication.

Earlier HSV replication initiation models were based on the observation that UL9 is able to bind two closely located binding sites within the OriS origin sequence. The model proposed at the time was that UL9 binding in OriS could cause the origin itself to loop in manner that would result in destabilization, leading to the opening of the origin and the initiation of HSV DNA replication. However, in the absence of an origin-dependent HSV DNA replication assay, this model has never been tested. The authors point out that if UL9 was capable of this type of OriS looping, this event would likely be observable using the single molecule approaches described in this manuscript. However, this is not observed. Instead, evidence of intermolecular interactions and additional binding sites on viral DNA are invoked. We suggest that several additional experiments would be required to present a convincing story about such a role of UL9 in HSV DNA replication. For instance, a recombination assay that shows that ATPase/helicase/DNA binding mutants of UL9 fail to stimulate recombination would be helpful. Several UL9 mutants have been reported that fail to hydrolyze ATP or fail to bind DNA. One recombination assay measures recombination directly between ts mutants. Even more informative and more approachable would be to use chromosomally integrated reporter assays developed by Maria Jasin and Jeremy Stark to measure various types of repair/recombination that occur in cells.

Specific Comments:

Introduction.

Line 45. "The HSV-1 genome consists of a 152 kb linear dsDNA within the viral capsid, which circularizes upon entry into the host nucleus through an unknown recombination mechanism". This statement reflects a lack of understanding of the current state of the field of HSV DNA replication. The literature review in the introduction and discussion are missing several key observations made in the field since the introduction of the rolling circle model for HSV DNA replication. Although rolling circle replication is the textbook version of the mechanism of DNA replication, it is not supported by current literature. The following observations directly contradict the notion of circularization.
1) Jackson and Deluca found that it was not possible to detect circular viral genomes in cells infected with wild type HSV-1. Circular genomes were only seen in cells infected with an ICP0 mutant. This is very compelling data.
2) Supporting evidence for circularization includes the disappearance of genome free ends and the detection of junctional fragments early in infection; however, recombination alone could explain the disappearance without invoking circularization.
3) If the HSV genome were to circularize, it would likely depend on one or more of the host DDR pathways to do so. The two most likely pathways are homologous recombination (HR) and NHEJ; however, components of both NHEJ and HR have been shown to be antiviral, and HSV has been shown to actively suppress them.
4) The immediate early protein ICP0 is involved in suppression of antiviral pathways by targeting DNA repair proteins for degradation. NHEJ proteins DNA-PK and HR proteins RNF8 and RNF168 are actively degraded by ICP0.
5) Using reporter assays, HSV infection stimulates SSA, while suppressing HRR and NHEJ.
6) Genomic inversions between unique regions of the genome (UL and US) occur as soon as DNA synthesis can be detected. This timing is not consistent with rolling circle replication.
Line 50. The origin-independent phase has been suggested to occur in a rolling-circle amplification mechanism, leading to the generation of large head-to-tail concatemers, which have been observed13,14. However, such concatemers could also arise from a recombination-dependent mechanism14–16. This needs to be rewritten for clarity
Line 67. Since the origins are located in dsDNA regions within the genome, UL9 helicase activity cannot explain the mechanism of replication initiation. This does not make any sense, given that UL9 binds to the origins. Furthermore, UL9 helicase mutants fail to rescue DNA replication, indicating a need for the helicase activity. Finally, OriS (and OriL) are located in areas of very active transcription. The unwinding or destabilization of this DNA by RNA polymerase could provide the necessary access to the origin.

Line 82. "We also found that UL9 has only a weak preference for the origin sequence and binds stably to other sequences in

mechanically stretched DNA". This is confusing and not convincing. Is this a property of specific sequences in the viral DNA? Can the authors identify the alternative binding sites? A mutant defective in origin binding would help inform this interpretation. Also see the specific comment for figure 4 below.

Line 84. "Furthermore, we show that UL9 tethers dsDNA inter-molecularly, suggesting a role during recombination". This is too strongly worded considering there is no evidence presented. An assay to monitor recombination is needed.
Line 87. "This model has been supported by previous observations of the high amount of recombination of HSV-1 genomes18,32". This is a very incomplete list of references that support recombination which is known to be a frequent event between two coinfecting viral genomes in cell culture, animal models and in circulating in patient populations.
Results:

Lines 98-100. "In the absence of UL9, we only observe trajectories at FRET 98 ~0.07, confirming that the DNA is extended (Fig. 1C and E and S1A). Addition of UL9 results in no apparent FRET efficiency increase for ~97% of observed molecules (N = 892)." It is not clear what Figure 1E is showing. The authors claim to only see 3% of molecules with FRET signal (1F), but 1E looks like they see a full spectrum of FRET. It is not clear that the 3 numbers above the dark grey histograms refer to. This figure is not described in the text either.

Lines 107-109. "Using Alternating Laser Excitation (ALEX)35, we can simultaneously monitor UL9 binding by direct acceptor emission (Fig. 2B-D, red) and approximate binding location by FRET" Figure 2 would be enhanced with a no-UL9 control, or a UL9 DNA binding mutant. The inclusion of a gel shift assay to show binding to this DNA substrate with strengthen the single molecule data.

Line 155-157. "Addition of ATP results in a comparable most-probable loop size of 390 ± 104 bp, but the non-hydrolyzable ATPγS increases it to 486 ± 182 bp, and both maximum loop sizes decrease below 1.5 kbp" Why are the loops larger with ATP? This figure needs an ATPase-deficient UL9 control.

Line 182-184. "At low stretching force (2pN), we see UL9 binding throughout cos6 DNA with a peak at the expected oriS location in the absence or presence of ATP (7.2 kbp, Fig. 4A and 183 S4E). However, this preference is lost at higher forces" This figure is based on the assumption that there is only one binding site for UL9 in the DNA, OriS. The conclusion that UL9 binds the entire DNA molecule at low force is from Figure S4A without ATP; however, at low force plus ATP, the binding to OriS looks very specific (S4E). It also looks like at high force (10pN) that there are 3 unique binding sites for UL9 in the presence of ATP (S4G). These experiments should be repeated with HSV DNA without an origin and with non-HSV DNA.

Line 196-198. "Addition of 4 nM UL9 led to an average loop number of 2.7 ± 1.3 (n = 59) per molecule in the absence of ATP, which did not change significantly (p = 0.157) in the presence of ATP" The ATP data is mentioned but not included in the figure. This figure would also be strengthened from the inclusion of non-OriS or non-HSV DNA control.

Line 814. (Figure 6 legend). "Example acceptor traces under direct acceptor excitation" It is not clear if B-D are showing acceptor or donor excitation. Legend says acceptor, picture says donor. Also, it is not clear if the peaks indicate transient binding.

Discussion:
Line 488. "Soon after infection and entry into the nucleus, the genome undergoes end recombination and circularizes8". Revise (see above)
Line 498. "Analogous to Redβ, the HSV-1 ssDNA binding protein ICP8 was shown to mediate ssDNA strand annealing as well as dsDNA strand invasion69,70." This last observation about strand invasion was retracted by the author. There is no evidence that strand invasion can occur in this system.
Line 500. "In bacteriophage λ, no helicase is required to mediate recombination. However, helicases such as HELQ in humans and Hel308 in archaea play a central role in DNA damage repair. In line with our observations for UL9, human repair proteins (e.g. Rad5271 and HELQ72) have also been found to tether DNA intermolecularly. Interestingly, Hel308 was suggested to play a central role as regulator of homologous recombination73. Due to its requirement during the first phase of replication but not the second, UL9 might also take up the role of a regulator74–76. Supporting our hypothesis that UL9 is involved in regulating HSV-mediated DNA recombination, it was previously shown that UL9 knock-down altered the recombination of adeno-associated virus genomes in an HSV-1 co-infection setup77. In conclusion, we show that UL9 efficiently and rapidly tethers origin- and non-origin dsDNA leading to the formation of static DNA loops with preferential sizes of about 300 to 500 bp and the ability to bend the origin at low efficiency." This paragraph should be removed. The link to Rad52 and HelQ is weak. These are DNA repair proteins that function in strand annealing and recombination. HelQ helicase activity is needed to remove RPA from ssDNA in order to promote annealing by Rad51/Rad52. This is a very different function than unwinding an origin.
Minor concerns:
Line 36. Retrieves. Perhaps authors meant to say retreats?
Line 40. HSV-1 is one of eight human pathogenic herpesviruses. It is more common to refer to the number of families of herpesviruses as nine.
Line 42. "Given the frequently severe outcomes (e.g., tumor formation) of herpesvirus infections in humans and animals, it is of great importance to understand their intricate replication mechanism." Tumor formation is not a frequent outcome for HSV (it rarely happens). This needs to be removed.

The authors should refer to the following manuscripts for more information about the apparent lack of circularization of the genome and the inhibition and antiviral nature of NHEJ and HR:

Jackson, S. A.; DeLuca, N. A. Relationship of herpes simplex virus genome configuration to productive and persistent infections. Proceedings of the National Academy of Sciences of the United States of America 100, 7871–7876 (2003). [PMID:12796511]
Schumacher, A.; Mohni, K.; Kan, Y.; Hendrickson, E.; Stark, J.; Weller, S. The HSV-1 Exonuclease, UL12, Stimulates Recombination by a Single Strand Annealing Mechanism. PLoS Pathogens 8, e1002862 (2012). [PMID:22912580]
Lees-Miller, S. P.; Long, M. C.; Kilvert, M. A.; Lam, V.; Rice, S. A.; Spencer, C. A. Attenuation of DNA-Dependent Protein Kinase Activity and Its Catalytic Subunit by the Herpes Simplex Virus Type 1 Transactivator ICP0. J of Virology 70, 7471–7477 (1996).
Parkinson, J.; Lees-Miller, S. P.; Everett, R. D. Herpes Simplex Virus Type 1 Immediate-Early Protein Vmw110 Induces the Proteasome-Dependent Degradation of the Catalytic Subunit of DNA-Dependent Protein Kinase. J of Virology 73, 650–657 (1999).
Lilley, C. E.; Chaurushiya, M. S.; Boutell, C.; Everett, R. D.; Weitzman, M. D. The Intrinsic Antiviral Defense to Incoming HSV-1 Genomes Includes Specific DNA Repair Proteins and Is Counteracted by the Viral Protein ICP0. PLoS Pathogens 7, e1002084 (2011).
Taylor, T. J.; Knipe, D. Proteomics of herpes simplex virus replication compartments: association of cellular DNA replication, repair, recombination, and chromatin remodeling proteins with ICP8. Journal of virology 78, 5856–5866 (2004). [PMID:15140983]
Lilley, C. E.; Schwartz, R. A.; Weitzman, M. D. Using or abusing: viruses and the cellular DNA damage response. Trends in Microbiology 15, 119–126 (2007). [PMID:17275307]

(Remarks on code availability)
I was not able to install and run the code.

Reviewer #3

(Remarks to the Author)
I co-reviewed this manuscript with one of the reviewers who provided the listed reports. This is part of the Nature Communications initiative to facilitate training in peer review and to provide appropriate recognition for Early Career Researchers who co-review manuscripts.

(Remarks on code availability)

Reviewer #4

(Remarks to the Author)
This manuscript presents an extremely compelling and technically rigorous investigation into the molecular role of the HSV-1 origin binding protein UL9 in DNA binding and potential replication initiation mechanisms. Using a sophisticated set of single-molecule approaches (smFRET, optical tweezers), the authors convincingly demonstrate that UL9 rarely induces intramolecular looping of oriS DNA, contrary to long-standing models, but instead forms efficient origin-independent loops and even inter-molecular DNA tethers. These findings challenge the classical origin looping model and point to an intriguing alternative role for UL9 in recombination-mediated replication. The data presented in this manuscript are extremely timely and of very high quality and outstanding interest to the fields of molecular biology, nucleic acid-protein interactions, and single-molecule biophysics. I am therefore happy to enthusiastically recommend publication of this manuscript, pending the following minor textual clarifications:

1. The low efficiency of oriS looping (~3%) relative to UL9-mediated DNA tethering (~30%) may partly reflect geometric or mechanical constraints of the short surface-immobilized DNA construct used in the smFRET assay. It would be useful if the authors could briefly comment on how surface tethering might influence their measured looping efficiency versus the more flexible tethering geometry in the intermolecular assay (between a surface-bound DNA and a freely diffusing DNA in solution).
2. The bi-phasic binding and unbinding kinetics are interpreted as reflecting different binding modes or sequence affinities. It would be helpful if the authors could expand a bit on the possible structural basis of these modes.
3. In the optical tweezers experiments, the authors show that UL9 exhibits some sequence preference for oriS at low force (2 pN), but this preference disappears at higher tension (>5 pN). This is an important observation - DNA tension affects UL9 binding behavior. However, in a cellular context, DNA is not necessarily under uniform tension, and it would be helpful if the authors could expand their discussion on the physiological relevance of the applied forces, i.e., to what extent 2-5 pN reflects conditions inside the nucleus.

(Remarks on code availability)

Version 1:

Reviewer comments:

Reviewer #1

(Remarks to the Author)
I would like to congratulate the authors for their detailed and precise answers to my queries, and I therefore fully recommend this article for publication.

(Remarks on code availability)

Reviewer #2

(Remarks to the Author)
This resubmitted manuscript entitled "HSV-1 origin binding protein UL9 forms intermolecular DNA tethers and intramolecular DNA loops" addresses most of the technical concerns raised by reviewers 2 and 3. Requested controls such as EMSA data and the inclusion of non-HSV DNA in the binding experiments were also included. However, serious problems remain regarding the presentation of background material. The introduction is still quire misleading and misses the points made in the first review. This indicates lack of understanding of the literature surrounding the mechanism of HSV DNA replication. In addition, the new description of the new lambda DNA sequences in the DNA binding experiments is not discussed accurately in the results, and their observations in the experiment are ignored in the text. Furthermore, the lack of inclusion of a UL9 mutant is troubling,
Specific comments:
1. Line 45-56. "The HSV-1 genome consists of a 152 kb linear dsDNA within the viral capsid, which is believed to circularize upon entry into the host nucleus through an unknown recombination mechanism 9–14."
These references are either irrelevant to the topic of circularization during lytic infection or misleading. Papers 12-14 describe the state of the genome during latent infection, and there is no controversy about the presence of circular genomes at that stage. These new references should be removed. Paper 10 relies on the loss of HSV DNA termini during lytic infection, which is not a reliable indication as the loss of termini could occur during recombination without the formation of circular genomes.
To say that there have been reports of circularization (and theta replication) is fine, but the way this section is written implies that this model has been accepted. Reasonable doubts have been raised, the most convincing being Jackson and Deluca who have shown that during lytic infection, circular genomes are not detected. Circular genomes are only detected in the absence of the viral immediate protein ICP0. A more balanced introduction of this topic is needed. The observations that the activities of ICP0 would be expected to prevent circularization requires comment by the authors.
2. Lines 47-48. "However, there is evidence suggesting that HSV-1 genome circularization may not be required during lytic replication and predominantly plays a role during latency15."
This statement is also misleading. The evidence is not that "circularization is not required". The evidence is that circularization may not occur. This is an important distinction.
3. Lines 50-52. "The origin-dependent phase is usually depicted in a theta (θ) amplification mechanism.
Although technically correct, this is also somewhat misleading. Something like "although original models proposed that origin dependent replication occurs by a theta model, no direct evidence for this mechanism has been found and other models have been proposed" would be more appropriate.
4. Lines 51-52, even though no direct evidence for this mechanism has been found17,18."
This statement is correct, but incomplete and confusing. It is odd that the authors have chosen to write this paper this way, since a recombination model actually supports their own data.
5. Lines213-219, Nevertheless, we can clearly observe prolonged binding to non-origin sequences under all tested conditions. Using λ DNA as (non-HSV-1 DNA) template, we can clearly see that UL9 also binds DNA that was not derived from HSV-1 (Supplementary Fig. 4I and J). Importantly, λ-DNA contains one 216 copy of "CGTTCGCACT", the BoxI sequence63,64, located ~20.2 kb from the center. In parallel 217 with other locations on the λ-DNA, UL9 exerts clear preference for the BoxI sequence 218 (Supplementary Fig. 4I and J).
This statement is still misleading about the data presented. Supplemental Fig 4I and 4J clearly show specific UL9 binding to three or four unique sequences along the length of the lambda genome. The authors reference the copy of BoxI sequences, but they ignore or do not comment on the other unique binding sites observed under both low and high pressure. These are partially addressed in the response to reviewer, but are essentially ignored by the author's in the text. These data should be discussed more fully.

6. It is troubling that the authors have not taken the reviewers' suggestions about using UL9 mutants. Many mutants have been expressed and purified. It is very straightforward these days to ask a company such as Azenta or others to carry out site directed mutagenesis and obtain an ATPase or DNA binding mutant. Their reasons for not doing this are not compelling. While the experiments with ATP and ATPgamma S are important, the inclusion of mutant proteins is essential to rule out that the observed activities are specific to UL9 and not a co-purifying DNA binding protein.

7. The authors were dismissive of the references and discussions about the role of ICP0. "All of these references refer to studies concerning ICP0-mediated modulation of the cellular DNA damage response, which is not directly connected to this study. While we appreciate the importance of the suggested references, we do not see a direct connection to the present study and have opted not to include them."

This is incorrect. They point out that they are not studying ICP0. What they miss here is that the presence of ICP0 has a direct correlation with the structure of the replicating DNA. Two direct lines of evidence are overlooked by the authors: (1)

Jackson and Deluca only observe circular genomes in the absence of ICP0; (2) ICP0 directly degrades the essential end joining protein DNA-PK. Together these data provide evidence that during a lytic infection with ICP0, there is ample evidence that circularization of the genome would be expected to be actively inhibited.

(Remarks on code availability)

Reviewer #3

(Remarks to the Author)
I co-reviewed this manuscript with one of the reviewers who provided the listed reports. This is part of the Nature Communications initiative to facilitate training in peer review and to provide appropriate recognition for Early Career Researchers who co-review manuscripts.

(Remarks on code availability)

Reviewer #4

(Remarks to the Author)
The authors have done an outstanding job answering the reviewers' comments. I therefore enthusiastically recommend publication.

(Remarks on code availability)

Version 2:

Reviewer comments:

Reviewer #2

(Remarks to the Author)
The authors have done a good job of addressing most of the comments made in the previous reviews. I have just one last correction that needs to be made for accuracy.

Line 97. “Recombination-mediated replication has emerged as a prevailing model for the initiation of HSV-1 genome replication (28,46,47), supported by extensive evidence of high levels of recombination in HSV-1 genomes in both cell culture and in vivo (31,48–52).”
It is misleading to suggest that recombination-mediated replication has emerged as a prevailing model for the initiation of HSV-1 genome replication. The models that are described in the papers cited (28, 46 and 47) actually show that recombination (specifically single strand annealing) promotes replication at existing replication forks. There is no evidence that recombination plays a role in initiation. Origin -dependent replication and recombination are considered to be distinct events in replication.

Reviewer #3

(Remarks to the Author)
I co-reviewed this manuscript with one of the reviewers who provided the listed reports. This is part of the Nature Communications initiative to facilitate training in peer review and to provide appropriate recognition for Early Career Researchers who co-review manuscripts.

Open Access This Peer Review File is licensed under a Creative Commons Attribution 4.0 International License, which permits use, sharing, adaptation, distribution and reproduction in any medium or format, as long as you give appropriate credit to the original author(s) and the source, provide a link to the Creative Commons license, and indicate if changes were made.
In cases where reviewers are anonymous, credit should be given to 'Anonymous Referee' and the source.
The images or other third party material in this Peer Review File are included in the article's Creative Commons license, unless indicated otherwise in a credit line to the material. If material is not included in the article's Creative Commons license and your intended use is not permitted by statutory regulation or exceeds the permitted use, you will need to obtain permission directly from the copyright holder.
To view a copy of this license, visit https://creativecommons.org/licenses/by/4.0/

REVIEWER COMMENTS

Reviewer #1:

Review for "HSV-1 origin binding protein UL9 forms intermolecular DNA tethers and intramolecular DNA loops"

In "HSV-1 origin binding protein UL9 forms intermolecular DNA tethers and intramolecular DNA loops", Meier and colleagues have investigated how UL9, a replication origin binding protein, associates with the two nearby origin sites and whether, upon binding, the DNA-associated UL9s form a loop. To this end, they use several single-molecule technologies (optical tweezers with fluorescence, TIRF-based FRET, flow stretching) to determine the mode of binding of UL9, its specificity and its ability to form DNA loops. Overall, the manuscript is well-structured with well-designed experiments that adequately answer the questions of the authors. The topic is new and important, given the prevalence of herpes virus, this new study will pave the way to future important work. I would therefore highly recommend it for publication.

We thank the reviewer for their kind comments.

I have some minor points that I would appreciate the authors fix before publication:

1- Figure 1D: The trace starts at high FRET and DNA looping by UL9 has equilibrated: can we have data where we observe this process dynamically, i.e. trace starting before adding UL9 and ending with a bent DNA in the presence of UL9? (the bottom right corner trace of Fig. S1 is a good example, though a single one).

To address this, we have included new FRET trajectories that show the process dynamically, starting with extended DNA (0 FRET), followed by DNA looping (excursions to higher FRET). We have now included this in Supplementary Figure S1D.

2- l. 158-164: the whole paragraph on how the gamma distribution scale parameter (Fig. 3F-H) describe the role of ATP needs to be better written and explain for the broad readership of Nature Communications. I am not familiar with the model of ref. 42, and I should not have to read this reference to understand how the authors use the model to reach their conclusion. Please describe rapidly the model and how you apply it. For example, I don't understand why an increase in the gamma distribution exponent says something about ATP binding and DNA looping. Also, the sentences in the paragraph are rather long.

We have rewritten the paragraph for clarity and added more references to protein-induced DNA loop formation and biopolymer physics (Page 8, Lines 178-195):

"Next, we assessed the loop-size distribution (**Fig. 3F – H**), which can be fit to a gamma distribution as previously described[1–4], defined by a scale (l_0) and a shape (n) factor (see Methods). The distribution profile arises from the fact that loops smaller than the persistence length of dsDNA (~150 bp) are unlikely to occur, while large loops are entropically unfavorable[5]. In the absence of ATP, the most probable loop length is 318 ± 71 bp, reaching maximum loop sizes of ~2 kbp (**Fig. 3F and I**). Addition of ATP or non-hydrolyzable analogue (ATPγS) results in longer most-probable loop sizes (390 ± 104 bp and 486 ± 182 bp, respectively) but shorter maximum loop sizes (~1.5 kbp, **Fig. 3G, H, J and K**). This result is in line with our earlier observation that the addition of ATP or ATPγS reduces the number of loops per molecule (**Fig. 3E**), since fewer longer loops can fit in a given length of DNA template[3]. In agreement with this, the shape factor in the absence of ATP (n = 2.4 ± 0.3) is significantly lower than in the presence of ATP (4.0 ± 0.6) or ATPγS (4.4 ± 0.9), indicating that

the addition of ATP or ATPγS restricts small loop formation. Along the same lines, the scale factor decreases in the presence of ATP or ATPγS from l_0 = 225 ± 27 bp (no ATP) to 128 ± 21 bp and 143 ± 30 bp, respectively, indicating less variability in loop size. Overall, both the increase in n and the decrease in l_0 indicate that the addition of ATP or ATPγS leads to more restrictive loop formation. The fact that ATP and ATPγS result in similar behaviors further suggests that ATP binding, but not hydrolysis, restricts loop formation."

3- In Figure 4, the authors show that UL9 binds more specifically at oriS at low force than at high force. Don't you think this result argue in favor of DNA bending by UL9 at the origin?

This is an interesting point. However, our TIRF-based looping data (Figure 1) show that UL9-induced looping at the origin under no force is very unlikely. Therefore, we do not believe that our data argues in favor of DNA bending by UL9 at the origin.

Minor comments:

4- In the abstract:

"the HSV-1 origin binding protein UL9 binds two closely located binding sites" a lot of binding here.

We have rephrased as (Page 2, Lines 22-22): "the HSV-1 origin binding protein UL9 interacts with two closely located sites".

5- l.66-67: "Despite being a superfamily II helicase, UL9 requires a 3' single-stranded DNA (ssDNA) overhang to mediate strand separation" Why despite being a SF2 helicase is in opposition to needing a 3' single-stranded DNA?

We agree with the reviewer. We have rephrased the manuscript as follows (Page 4, Line 70):

"UL9 is a superfamily II helicase and requires a 3' single-stranded DNA (ssDNA) overhang to mediate strand separation."

6- l.76-77 "and access to the ssDNA to other replication factors and replisome assembly" I would rather re-write this as: "and enable other replication factors to access the ssDNA and subsequent replisome assembly"

We agree with the suggested changes and have modified the manuscript accordingly (Page 4, Lines 85-86).

7- l.79: "which eliminate ensemble averaging": in your case, more importantly, the ability to manipulate and observe the DNA molecule and capture such looping is the most important, isn't it?

We appreciate the reviewer's comment and edited as follows (Page 5, Lines 91-93):

"Importantly, force spectroscopy (optical tweezers) allows us to directly manipulate DNA molecules and measure changes in force, enabling us to gain a detailed understanding of how UL9 interacts with DNA."

8- l.217-218: you use the verb "tethers": I think binds or attaches is more adapted in this context. Same l.471.

We appreciate the reviewer’s comment, but in this context, tethering is the correct terminology, as previously used in the context of Cohesin tethering two DNA strands (Gutierrez-Escribano *et al.* (2019) Sci Adv 5:eaay6804, PMID: 31807710).

Reviewer #2:

This manuscript entitled "HSV-1 origin binding protein UL9 forms intermolecular DNA tethers and intramolecular DNA loops" describes a novel previously unrecognized behavior for the HSV origin binding protein, UL9. The authors present data suggesting that UL9 can tether two OriS DNA molecules intermolecularly, forming large DNA loops. This surprising result is of considerable interest as it is possible that intermolecular interactions between separate genomes could lead to recombination events that could alter models for HSV DNA replication. The actual data presented are for the most part of high quality and support interpretation that intermolecular interactions can occur. However, the background material presented is incomplete and relies heavily on a outdated model suggesting that the HSV genome circularizes during replication and replicates by rolling circle replication. Considerable evidence has been published over the last 20 years that indicate that HSV DNA replication is much more complex. The manuscript as written contains misleading and incompletely referenced statements. Thus, although the major finding of this paper of long-distance intermolecular looping is extremely interesting, major revision as well as additional experiments will need to be addressed before this manuscript is suitable for publication.

Earlier HSV replication initiation models were based on the observation that UL9 is able to bind two closely located binding sites within the OriS origin sequence. The model proposed at the time was that UL9 binding in OriS could cause the origin itself to loop in manner that would result in destabilization, leading to the opening of the origin and the initiation of HSV DNA replication. However, in the absence of an origin-dependent HSV DNA replication assay, this model has never been tested. The authors point out that if UL9 was capable of this type of OriS looping, this event would likely be observable using the single molecule approaches described in this manuscript. However, this is not observed. Instead, evidence of intermolecular interactions and additional binding sites on viral DNA are invoked. We suggest that several additional experiments would be required to present a convincing story about such a role of UL9 in HSV DNA replication. For instance, a recombination assay that shows that ATPase/helicase/DNA binding mutants of UL9 fail to stimulate recombination would be helpful. Several UL9 mutants have been reported that fail to hydrolyze ATP or fail to bind DNA. One recombination assay measures recombination directly between ts mutants. Even more informative and more approachable would be to use chromosomally integrated reporter assays developed by Maria Jasin and Jeremy Stark to measure various types of repair/recombination that occur in cells.

We appreciate the reviewer's thoughtful comments. In the specific comments section below, we clarify these points and address the additional experiments suggested here.

Specific Comments:

Introduction.

Line 45. "The HSV-1 genome consists of a 152 kb linear dsDNA within the viral capsid, which circularizes upon entry into the host nucleus through an unknown recombination mechanism". This statement reflects a lack of understanding of the current state of the field of HSV DNA replication. The literature review in the introduction and discussion are missing several key observations made in the field since the introduction of the rolling circle model for HSV DNA replication. Although rolling circle replication is the textbook version of the mechanism of DNA replication, it is not supported by current literature. The following observations directly contradict the notion of circularization.
1) Jackson and Deluca found that it was not possible to detect circular viral genomes in cells infected with wild type HSV-1. Circular genomes were only seen in cells infected with an

ICP0 mutant. This is very compelling data.
2) Supporting evidence for circularization includes the disappearance of genome free ends and the detection of junctional fragments early in infection; however, recombination alone could explain the disappearance without invoking circularization.
3) If the HSV genome were to circularize, it would likely depend on one or more of the host DDR pathways to do so. The two most likely pathways are homologous recombination (HR) and NHEJ; however, components of both NHEJ and HR have been shown to be antiviral, and HSV has been shown to actively suppress them.
4) The immediate early protein ICP0 is involved in suppression of antiviral pathways by targeting DNA repair proteins for degradation. NHEJ proteins DNA-PK and HR proteins RNF8 and RNF168 are actively degraded by ICP0.
5) Using reporter assays, HSV infection stimulates SSA, while suppressing HRR and NHEJ.
6) Genomic inversions between unique regions of the genome (UL and US) occur as soon as DNA synthesis can be detected. This timing is not consistent with rolling circle replication.

We are aware of the ongoing debate about whether or not HSV-1 genomes circularize upon entry into the nucleus. However, genome circularization upon entry is not a question addressed in this manuscript. Nonetheless, we recognize the importance of stimulating engagement with different hypotheses. Therefore, to address the reviewer's concern, we have included the publication suggested by the reviewer (Jackson/De Luca) alongside additional papers, which support the alternative hypothesis (Strang/ Stow, Poffenberger/ Roizman, Garber/Beverley/Cohen, Efstathiou, Mellerick, Rock), and edited the manuscript accordingly (Page 3, Lines 46-49).

"The HSV-1 genome consists of a 152 kb linear dsDNA within the viral capsid, which is believed to circularize upon entry into the host nucleus through an unknown recombination mechanism[6–11]. However, there is evidence suggesting that HSV-1 genome circularization may not be required during lytic replication and predominantly plays a role during latency[12]."

Line 50. The origin-independent phase has been suggested to occur in a rolling-circle amplification mechanism, leading to the generation of large head-to-tail concatemers, which have been observed13,14. However, such concatemers could also arise from a recombination-dependent mechanism14–16. This needs to be rewritten for clarity

To clarify this statement, we have rewritten it as follows (Page 3, Lines 54-56):

"The origin-independent phase has been suggested to occur in a rolling-circle amplification (RCA) mechanism. The product of RCA is expected to be large head-to-tail concatemers, which have been observed for HSV-1 replication[13,14]. However, such concatemers could also arise from a recombination-dependent mechanism[14–16]."

Line 67. Since the origins are located in dsDNA regions within the genome, UL9 helicase activity cannot explain the mechanism of replication initiation. This does not make any sense, given that UL9 binds to the origins. Furthermore, UL9 helicase mutants fail to rescue DNA replication, indicating a need for the helicase activity. Finally, OriS (and OriL) are located in areas of very active transcription. The unwinding or destabilization of this DNA by RNA polymerase could provide the necessary access to the origin.

We agree with the reviewer that the original sentence did not capture our intended message clearly. To address this comment, we have rephrased the text to emphasize the missing information on the importance of UL9 helicase activity (Page 4, Lines 71-76):

"Therefore, the UL9 helicase activity alone cannot mediate unwinding of the origins, which are located in dsDNA regions of the genome. It is possible that active transcription by RNA

polymerase provides access to the origins. Although mutants in the helicase motifs fail to support HSV-1 replication, the precise role of UL9 during replication initiation remains unclear. To investigate how UL9 interacts with DNA, we used single-molecule microscopy."

Line 82. "We also found that UL9 has only a weak preference for the origin sequence and binds stably to other sequences in mechanically stretched DNA". This is confusing and not convincing. Is this a property of specific sequences in the viral DNA? Can the authors identify the alternative binding sites? A mutant defective in origin binding would help inform this interpretation. Also see the specific comment for figure 4 below.

To address this concern, we have performed additional optical tweezers experiments with a non-HSV-1 control DNA (λ-DNA). The new data (**Supplementary Figure 4I and J**) confirm that UL9 binds to the control DNA, which is unrelated to HSV-1. Together with the data in the original submission, these results confirm that UL9 can bind to non-origin sequences, in agreement with previous results (Hammarsten, 1996), as outlined in the discussion.

Apart from the origin sequence, whose location is well-defined, the diffraction-limited experimental resolution (~100 nm or 300 bp) prevents us from identifying binding sequences. Regarding origin-binding defective mutants, they are heavily truncated variants of UL9 that have never been purified beyond cell extracts, making it too challenging for us to test on our optical tweezers setup.

Line 84. "Furthermore, we show that UL9 tethers dsDNA inter-molecularly, suggesting a role during recombination". This is too strongly worded considering there is no evidence presented. An assay to monitor recombination is needed.

To soften this statement, we have reworded it as follows (Page 5, Lines 97-99):

"Furthermore, we show that UL9 tethers dsDNA inter-molecularly, raising the possibility that UL9 might play a role during recombination."

Line 87. "This model has been supported by previous observations of the high amount of recombination of HSV-1 genomes18,32". This is a very incomplete list of references that support recombination which is known to be a frequent event between two coinfecting viral genomes in cell culture, animal models and in circulating in patient populations.

As requested, we have extended the list of references to include additional *in vivo* studies (Page 5, Lines 100-102):

"This model has been supported by previous observations of the high amount of recombination of HSV-1 genomes in cell cultures and *in vivo*[12,19–24]."

Results:

Lines 98-100. "In the absence of UL9, we only observe trajectories at FRET 98 ~0.07, confirming that the DNA is extended (Fig. 1C and E and S1A). Addition of UL9 results in no apparent FRET efficiency increase for ~97% of observed molecules (N = 892)." It is not clear what Figure 1E is showing. The authors claim to only see 3% of molecules with FRET signal (1F), but 1E looks like they see a full spectrum of FRET. It is not clear that the 3 numbers above the dark grey histograms refer to. This figure is not described in the text either.

We agree with the reviewer that this figure can be unclear to non-expert readers. The issue is that the dark histogram in Figure 1E contains only data from molecules that show FRET above the DNA-only peak (light grey distribution) at some point during the trajectory. This

means that 97% of the trajectories in the presence of UL9 were not included in the histogram, because the DNA-only peak would dominate the distribution. To clarify this, we have rewritten as follows (Page 5, Lines 112-118) and caption of Figure 1.

"In the absence of UL9, we only observe trajectories at no (~0 FRET efficiency) or low FRET (~0.07 FRET efficiency, **Fig. 1E and Supplementary Fig. 1A**), confirming that the DNA is extended (**Fig. 1C and E and Supplementary Fig. 1C**). Addition of UL9 results in no apparent FRET efficiency increase for ~97% of observed molecules (N = 892, **Supplementary Figure 1B**). Only a small fraction (~3%, **Fig. 1F**) exhibits FRET increases between 0.3 and 1 (**Fig.1D – F and Supplementary Fig. 1B and D**), consistent with UL9-induced DNA looping."

"**E** Histogram of FRET efficiencies in the absence (light grey) or presence of 20 nM UL9 (dark grey). Molecules showing no FRET were not included. In the presence of UL9 (dark grey), 97% of trajectories behaved as DNA-only and were not included to highlight the distribution of looping molecules (3%)."

Lines 107-109. "Using Alternating Laser Excitation (ALEX)35, we can simultaneously monitor UL9 binding by direct acceptor emission (Fig. 2B-D, red) and approximate binding location by FRET" Figure 2 would be enhanced with a no-UL9 control, or a UL9 DNA binding mutant. The inclusion of a gel shift assay to show binding to this DNA substrate with strengthen the single molecule data.

As suggested by the reviewer, we have now added two EMSAs (Supplementary Fig. 2A–C), as well as additional FRET trajectories without protein (DNA only; Supplementary Fig. 2G) and with UL9 (+/- ATP) (Supplementary Fig. 2H–J) to the manuscript. The new data confirm our initial findings. We have also modified the manuscript accordingly (Page 6, Lines 121-123):

"To confirm that UL9 binds the oriS DNA, we performed electrophoretic mobility shift assays (EMSAs), which reveal an apparent $K_{1/2}$ = 13 ± 1 nM and n = 1.9 ± 0.2 (**Supplementary Fig. 2A – C**)."

Line 155-157. "Addition of ATP results in a comparable most-probable loop size of 390 ± 104 bp, but the non-hydrolyzable ATPγS increases it to 486 ± 182 bp, and both maximum loop sizes decrease below 1.5 kbp" Why are the loops larger with ATP? This figure needs an ATPase-deficient UL9 control.

We have rewritten this paragraph for clarity, including a likely explanation for why we observe larger loops in the presence of ATP/ ATPγS and added more references to theoretical work on protein-induced DNA loop formation and biopolymer physics (Page 8, Lines 178-195):

"Next, we assessed the loop-size distribution (**Fig. 3F – H**), which can be fit to a gamma distribution as previously described[1–4], defined by a scale (l_0) and a shape (n) factor (see Methods). The distribution profile arises from the fact that loops smaller than the persistence length of dsDNA (~150 bp) are unlikely to occur, while large loops are entropically unfavorable[5]. In the absence of ATP, the most probable loop length is 318 ± 71 bp, reaching maximum loop sizes of ~2 kbp (**Fig. 3F and I**). Addition of ATP or non-hydrolyzable analogue (ATPγS) results in longer most-probable loop sizes (390 ± 104 bp and 486 ± 182 bp, respectively) but shorter maximum loop sizes (~1.5 kbp, **Fig. 3G, H, J and K**). This result is in line with our earlier observation that the addition of ATP or ATPγS reduces the number of loops per molecule (**Fig. 3E**), since fewer longer loops can fit in a given length of DNA template[3]. In agreement with this, the shape factor in the absence of ATP (n = 2.4 ± 0.3) is significantly lower than in the presence of ATP (4.0 ± 0.6) or ATPγS (4.4 ± 0.9), indicating that

the addition of ATP or ATPγS restricts small loop formation. Along the same lines, the scale factor decreases in the presence of ATP or ATPγS from l_0 = 225 ± 27 bp (no ATP) to 128 ± 21 bp and 143 ± 30 bp, respectively, indicating less variability in loop size. Overall, both the increase in *n* and the decrease in l_0 indicate that the addition of ATP or ATPγS leads to more restrictive loop formation. The fact that ATP and ATPγS result in similar behaviors further suggests that ATP binding, but not hydrolysis, restricts loop formation."
Given the consistency between the ATP and ATPγS data, experiments with an ATPase-deficient mutant are unlikely to yield new insights into the mechanism.

Line 182-184. "At low stretching force (2pN), we see UL9 binding throughout cos6 DNA with a peak at the expected oriS location in the absence or presence of ATP (7.2 kbp, Fig. 4A and 183 S4E). However, this preference is lost at higher forces" This figure is based on the assumption that there is only one binding site for UL9 in the DNA, OriS. The conclusion that UL9 binds the entire DNA molecule at low force is from Figure S4A without ATP; however, at low force plus ATP, the binding to OriS looks very specific (S4E). It also looks like at high force (10pN) that there are 3 unique binding sites for UL9 in the presence of ATP (S4G). These experiments should be repeated with HSV DNA without an origin and with non-HSV DNA.

For the C-trap experiments, we used a template DNA (cos6), which contains a single origin of replication (oriS) binding site, as described in the text:

157-159 "To this aim, we prepared a DNA substrate (cos6, 47.6 kb, see Methods) encoding about a third of the HSV-1 genome and containing a single origin of replication (oriS)."

174 "Since the cos6 DNA template contains a single origin of replication,…"

232-224 "Since UL9 appears to form oriS-independent loops, we sought to test whether UL9 has any sequence-specificity on the cos6 DNA, which contains a single oriS sequence located 7.2 kbp from the center of the linearized substrate (**Fig. 4**)."

However, the previous data in Supplementary Fig. 4E were somewhat misleading because of the low number of molecules analyzed (6 at 2 pN and 4 at 20 pN) in the presence of ATP. To address this, we have collected additional data at both 2 pN (n = 73) and 20 pN (n = 36) in the presence of ATP. The expanded dataset confirms the previously observed binding preference of UL9 to oriS at 2 pN remained, while more binding to non-oriS sequences was observed at 20 pN. We have also highlighted the expected location of the previously identified UL9 binding site within UL9 ORF ('UL9 box') within cos6. Finally, we have collected new data using λ-DNA, which is not derived from HSV-1 but contains a single BoxI sequence (**Supplementary Fig. 4I and J**).

We have adapted the revised manuscript accordingly (Page 10, Lines 243-254):

"However, this preference is lost at higher forces in the absence of ATP (>5 pN, **Fig. 4B and Supplementary Fig. 4**). Interestingly, the binding preference for oriS seems to be more pronounced in the presence of ATP even at higher forces (**Supplementary Fig. 4**). Furthermore, cos6 also contains the high-affinity binding site[25] within the UL9 ORF ('UL9 box'; TGTTCGCACT) at 11.2 kb from the middle, to which UL9 only seems to preferentially bind in the presence of ATP at 10 pN (**Supplementary Fig. 4G**). Nevertheless, we can clearly observe prolonged binding to non-origin sequences under all tested conditions. Using λ-DNA as (non-HSV-1 DNA) template, we can clearly see that UL9 also binds DNA that was not derived from HSV-1 (**Supplementary Fig. 4I and J**). Importantly, λ-DNA contains one

copy of "CGTTCGCACT", the BoxI sequence[26,27], located ~20.2 kb from the center. In parallel with other locations on the λ-DNA, UL9 exerts clear preference for the BoxI sequence (**Supplementary Fig. 4I and J**)."

At 10 pN + ATP, three specific locations appear to bind more prominently at the expected oriS location (~7 kb) and two additional locations (~12 and ~19 kb). However, since the latter two are not observed under any other condition, their significance is not clear.

Line 196-198. "Addition of 4 nM UL9 led to an average loop number of 2.7 ± 1.3 (n = 59) per molecule in the absence of ATP, which did not change significantly (p = 0.157) in the presence of ATP" The ATP data is mentioned but not included in the figure. This figure would also be strengthened from the inclusion of non-OriS or non-HSV DNA control.

We have now added the quantification of the loop numbers for the TIRF-based double-tethering experiments to **Supplementary Figure** 5E and referred to it in the manuscript. In the previous and the current versions of the manuscript, a movie of cos6 DNA in the presence of UL9 and ATP is shown (**Movie S2**).

As for non-HSV-1 DNA data, this was already included in the previous version of the manuscript, but we now highlighted this in the text (Page 11, Lines 274-276):

"In agreement with the optical tweezers experiments, we also observed HSV-1 DNA-independent looping using λ-DNA (**Supplementary Fig. 5C and D**)."

Line 814. (Figure 6 legend). "Example acceptor traces under direct acceptor excitation" It is not clear if B-D are showing acceptor or donor excitation. Legend says acceptor, picture says donor. Also, it is not clear if the peaks indicate transient binding.

We appreciate the reviewer's comment, as it appears we did mislabel the figure. The traces in Figure 6B-D show acceptor (DNA-Cy5) signal under acceptor excitation. We have now corrected accordingly.

Discussion:

Line 488. "Soon after infection and entry into the nucleus, the genome undergoes end recombination and circularizes8". Revise (see above)

We've now rewritten this section accordingly (Page 24, Lines 611-614):

"Soon after infection and entry into the nucleus, the number of genome ends have been shown to decrease[9,11], indicating that end recombination is taking place, which has previously been suggested to lead to circularization[6–8], concatemerization[14,28] or formation of branched DNA structures[29]."

Line 498. "Analogous to Redβ, the HSV-1 ssDNA binding protein ICP8 was shown to mediate ssDNA strand annealing as well as dsDNA strand invasion69,70." This last observation about strand invasion was retracted by the author. There is no evidence that strand invasion can occur in this system.

We would like to thank the reviewer for this valuable comment, as we had not been aware of the retraction of this observation. We have now removed the claim that ICP8 promotes strand annealing from the manuscript and have added additional references (Page 24, Lines 623-626):

"Analogous to Redβ, the HSV-1 ssDNA binding protein ICP8 was shown to mediate ssDNA strand annealing[30–34]."

Line 500. “In bacteriophage λ, no helicase is required to mediate recombination. However, helicases such as HELQ in humans and Hel308 in archaea play a central role in DNA damage repair. In line with our observations for UL9, human repair proteins (e.g. Rad5271 and HELQ72) have also been found to tether DNA intermolecularly. Interestingly, Hel308 was suggested to play a central role as regulator of homologous recombination73. Due to its requirement during the first phase of replication but not the second, UL9 might also take up the role of a regulator74–76. Supporting our hypothesis that UL9 is involved in regulating HSV-mediated DNA recombination, it was previously shown that UL9 knock-down altered the recombination of adeno-associated virus genomes in an HSV-1 co-infection setup77. In conclusion, we show that UL9 efficiently and rapidly tethers origin- and non-origin dsDNA leading to the formation of static DNA loops with preferential sizes of about 300 to 500 bp and the ability to bend the origin at low efficiency.” This paragraph should be removed. The link to Rad52 and HelQ is weak. These are DNA repair proteins that function in strand annealing and recombination. HelQ helicase activity is needed to remove RPA from ssDNA in order to promote annealing by Rad51/Rad52. This is a very different function than unwinding an origin.

As suggested, we have now removed the section discussing the parallels between UL9 and HELQ/ Hel308.

Minor concerns:

Line 36. Retrieves. Perhaps authors meant to say retreats?

We’ve modified the manuscript accordingly.

Line 40. HSV-1 is one of eight human pathogenic herpesviruses. It is more common to refer to the number of families of herpesviruses as nine.

There is only one family of herpesviruses that infects humans (*Orthoherpesviridae,* formerly *Herpesviridae*, order of *Herpesvirales*). We believe that the reviewer refers to the number of species that infect humans, of which there are indeed 9. In our manuscript, we’ve referred to the human pathogenic herpesvirus species, for which we did not count HHV6A and HHV6B as separate species since they were previously classified as variants of HHV6. However, we acknowledge that they’ve now been officially classified by the ICTV as separate species and adjusted the manuscript accordingly (Page 3, Lines 40-42):

“HSV-1 is one of nine human pathogenic herpesvirus species and, therefore, shares many features with other herpesviruses such as varicella-zoster virus, Kaposi sarcoma herpesvirus, and Epstein-Barr virus”

Line 42. “Given the frequently severe outcomes (e.g., tumor formation) of herpesvirus infections in humans and animals, it is of great importance to understand their intricate replication mechanism.” Tumor formation is not a frequent outcome for HSV (it rarely happens). This needs to be removed.

We agree with the reviewer’s comment and have changed the manuscript accordingly.

The authors should refer to the following manuscripts for more information about the apparent lack of circularization of the genome and the inhibition and antiviral nature of NHEJ and HR:

Jackson, S. A.; DeLuca, N. A. Relationship of herpes simplex virus genome configuration to productive and persistent infections. Proceedings of the National Academy of Sciences of the United States of America 100, 7871–7876 (2003). [PMID:12796511]

We have now included this reference.

Schumacher, A.; Mohni, K.; Kan, Y.; Hendrickson, E.; Stark, J.; Weller, S. The HSV-1 Exonuclease, UL12, Stimulates Recombination by a Single Strand Annealing Mechanism. PLoS Pathogens 8, e1002862 (2012). [PMID:22912580]

This reference was already included in the previous version of the manuscript.

Lees-Miller, S. P.; Long, M. C.; Kilvert, M. A.; Lam, V.; Rice, S. A.; Spencer, C. A. Attenuation of DNA-Dependent Protein Kinase Activity and Its Catalytic Subunit by the Herpes Simplex Virus Type 1 Transactivator ICP0. J of Virology 70, 7471–7477 (1996).
Parkinson, J.; Lees-Miller, S. P.; Everett, R. D. Herpes Simplex Virus Type 1 Immediate-Early Protein Vmw110 Induces the Proteasome-Dependent Degradation of the Catalytic Subunit of DNA-Dependent Protein Kinase. J of Virology 73, 650–657 (1999).
Lilley, C. E.; Chaurushiya, M. S.; Boutell, C.; Everett, R. D.; Weitzman, M. D. The Intrinsic Antiviral Defense to Incoming HSV-1 Genomes Includes Specific DNA Repair Proteins and Is Counteracted by the Viral Protein ICP0. PLoS Pathogens 7, e1002084 (2011).

All of these references refer to studies concerning ICP0-mediated modulation of the cellular DNA damage response, which is not directly connected to this study. While we appreciate the importance of the suggested references, we do not see a direct connection to the present study and have opted not to include them.

Taylor, T. J.; Knipe, D. Proteomics of herpes simplex virus replication compartments: association of cellular DNA replication, repair, recombination, and chromatin remodeling proteins with ICP8. Journal of virology 78, 5856–5866 (2004). [PMID:15140983]
Lilley, C. E.; Schwartz, R. A.; Weitzman, M. D. Using or abusing: viruses and the cellular DNA damage response. Trends in Microbiology 15, 119–126 (2007). [PMID:17275307]

Since these references refer to the viral modulation of and interaction with the cellular DNA damage protein, which is not within the scope of the presented study, we did not include them.

Reviewer #2 (Remarks on code availability):

I was not able to install and run the code.

We regret that the reviewer was not able to run the Code Ocean capsules. It works in our hands, so we are unsure about what caused the issue. For one of the capsules (generate kymographs from TIRF data), it would be required to launch the python cloud workstation before the code can be tested. The code is also deposited on the lab's GitHub page (https://github.com/singlemoleculegroup).

Reviewer #3:

I co-reviewed this manuscript with one of the reviewers who provided the listed reports. This is part of the Nature Communications initiative to facilitate training in peer review and to provide appropriate recognition for Early Career Researchers who co-review manuscripts.

Reviewer #4:

This manuscript presents an extremely compelling and technically rigorous investigation into the molecular role of the HSV-1 origin binding protein UL9 in DNA binding and potential replication initiation mechanisms. Using a sophisticated set of single-molecule approaches (smFRET, optical tweezers), the authors convincingly demonstrate that UL9 rarely induces intramolecular looping of oriS DNA, contrary to long-standing models, but instead forms efficient origin-independent loops and even inter-molecular DNA tethers. These findings challenge the classical origin looping model and point to an intriguing alternative role for UL9 in recombination-mediated replication. The data presented in this manuscript are extremely timely and of very high quality and outstanding interest to the fields of molecular biology, nucleic acid-protein interactions, and single-molecule biophysics. I am therefore happy to enthusiastically recommend publication of this manuscript, pending the following minor textual clarifications:

We thank the reviewer for their kind comments.

1. The low efficiency of oriS looping (~3%) relative to UL9-mediated DNA tethering (~30%) may partly reflect geometric or mechanical constraints of the short surface-immobilized DNA construct used in the smFRET assay. It would be useful if the authors could briefly comment on how surface tethering might influence their measured looping efficiency versus the more flexible tethering geometry in the intermolecular assay (between a surface-bound DNA and a freely diffusing DNA in solution).

The reviewer makes a fair point. To address this, we have edited the manuscript accordingly (Page 22, Lines 570-576):

"A possible explanation for the low efficiency of oriS looping is that surface tethering might influence the observed looping efficiency because of geometric or mechanical constraints of the short surface-immobilized DNA construct used in the smFRET assay. But we don't think this is a likely explanation because (i) the binding affinities measured by EMSA and smFRET are comparable (131 ± 70 and 32 ± 1 nM, respectively, **Supplementary Fig. 2**), and (ii) loops shorter than 100 bp are not frequently observed in the flexible tethering geometry of the optical tweezers experiments (**Figure 3F - H**)."

2. The bi-phasic binding and unbinding kinetics are interpreted as reflecting different binding modes or sequence affinities. It would be helpful if the authors could expand a bit on the possible structural basis of these modes.

The reviewer raises an interesting point. To address this, we are in the process of obtaining a cryoEM structure of UL9 bound to OriS DNA. The data is too preliminary to be included in this manuscript, but it already points at flexible backbone contacts between UL9 and the DNA, consistent with the idea of multiple, non-sequence-specific interaction modes, and in agreement with the multi-phasic unbinding kinetics observed here. We believe this structure is outside the scope of this manuscript, but will be the subject of a follow-up paper soon.

3. In the optical tweezers experiments, the authors show that UL9 exhibits some sequence preference for oriS at low force (2 pN), but this preference disappears at higher tension (>5 pN). This is an important observation - DNA tension affects UL9 binding behavior. However, in a cellular context, DNA is not necessarily under uniform tension, and it would be helpful if the authors could expand their discussion on the physiological relevance of the applied forces, i.e., to what extent 2-5 pN reflects conditions inside the nucleus.

This is certainly an interesting thought. To address this, we modified the manuscript as follows (Page 23, Lines 594-599):

“Interestingly, the force-sensitivity of origin-binding specificity observed here could indicate the presence of a force-dependent mechanism of UL9 regulation. UL9 is only required during the first 6 hours post-infection[35,36] and is even inhibitory if over-expressed[37–39]. Forces applied during DNA replication, for example, could be acting as mechano-regulators of UL9 activity. Indeed, several polymerases have been shown to function in this force regime[40–44].”

References:

1. Sankararaman, S. & Marko, J. F. Formation of loops in DNA under tension. *Phys. Rev. E* **71**, 021911 (2005).
2. Gemmen, G. J. DNA looping by two-site restriction endonucleases: heterogeneous probability distributions for loop size and unbinding force. *Nucleic Acids Res.* **34**, 2864–2877 (2006).
3. Sankararaman, S. & Marko, J. F. Entropic Compression of Interacting DNA Loops. *Phys. Rev. Lett.* **95**, 078104 (2005).
4. Myong, S., Bruno, M. M., Pyle, A. M. & Ha, T. Spring-Loaded Mechanism of DNA Unwinding by Hepatitis C Virus NS3 Helicase. *Science* **317**, 513–516 (2007).
5. Shimada, J. & Yamakawa, H. Ring-closure probabilities for twisted wormlike chains. Application to DNA. *Macromolecules* **17**, 689–698 (1984).
6. Garber, D. A., Beverley, S. M. & Coen, D. M. Demonstration of circularization of herpes simplex virus DNA following infection using pulsed field gel electrophoresis. *Virology* 459–62 (1993) doi:10.1006/viro.1993.1612.
7. Poffenberger, K. L. & Roizman, B. A noninverting genome of a viable herpes simplex virus 1: presence of head-to-tail linkages in packaged genomes and requirements for circularization after infection. *J. Virol.* **53**, 587–595 (1985).
8. Strang, B. L. & Stow, N. D. Circularization of the Herpes Simplex Virus Type 1 Genome upon Lytic Infection. *J. Virol.* **79**, 12487–12494 (2005).
9. Efstathiou, S., Minson, A. C., Field, H. J., Anderson, J. R. & Wildy, P. Detection of herpes simplex virus-specific DNA sequences in latently infected mice and in humans. *J. Virol.* **57**, 446–455 (1986).
10. Mellerick, D. M. & Fraser', N. W. Physical state of the latent herpes simplex virus genome in a mouse model system: Evidence suggesting an episomal state. *Virology* **158**, 265–275 (1987).
11. Rock, D. L. & Fraser, N. W. Detection of HSV-1 genome in central nervous system of latently infected mice. *Nature* **302**, 523–525 (1983).

12. Jackson, S. A. & DeLuca, N. A. Relationship of herpes simplex virus genome configuration to productive and persistent infections. *Proc. Natl. Acad. Sci.* **100**, 7871–7876 (2003).

13. Jacob, R. J., Morse, L. S. & Roizman, B. Anatomy of Herpes Simplex Virus DNA XII. Accumulation of Head-to-Tail Concatemers in Nuclei of Infected Cells and Their Role in the Generation of the Four Isomeric Arrangements of Viral DNA. *J. Virol.* **29**, 448–457 (1979).

14. Bataille, D. & Epstein, A. L. Herpes simplex virus replicative concatemers contain L components in inverted orientation. *Virology* (1994) doi:10.1006/viro.1994.1498.

15. Weerasooriya, S., DiScipio, K. A., Darwish, A. S., Bai, P. & Weller, S. K. Herpes simplex virus 1 ICP8 mutant lacking annealing activity is deficient for viral DNA replication. *Proc. Natl. Acad. Sci.* **116**, 1033–1042 (2019).

16. Schumacher, A. J. *et al.* The HSV-1 Exonuclease, UL12, Stimulates Recombination by a Single Strand Annealing Mechanism. *PLoS Pathog.* **8**, e1002862 (2012).

17. Martinez, R., Shao, L. & Weller, S. K. The conserved helicase motifs of the herpes simplex virus type 1 origin-binding protein UL9 are important for function. *J. Virol.* **66**, 6735–6746 (1992).

18. Marintcheva, B. & Weller, S. K. Residues within the Conserved Helicase Motifs of UL9, the Origin-binding Protein of Herpes Simplex Virus-1, Are Essential for Helicase Activity but Not for Dimerization or Origin Binding Activity. *J. Biol. Chem.* **276**, 6605–6615 (2001).

19. Hayward, G. S., Jacob, R. J., Wadsworth, S. C. & Roizman, B. Anatomy of herpes simplex virus DNA: evidence for four populations of molecules that differ in the relative orientations of their long and short components. *Proc. Natl. Acad. Sci.* **72**, 4243–4247 (1975).

20. Schaffer, P. A., Tevethia, M. J. & Benyesh-Melnick, M. Recombination between temperature-sensitive mutants of herpes simplex virus type 1. *Virology* **58**, 219–228 (1974).

21. Sarisky, R. T. & Weber, P. C. Requirement for double-strand breaks but not for specific DNA sequences in herpes simplex virus type 1 genome isomerization events. *J. Virol.* **68**, 34–47 (1994).
22. Bowden, R., Sakaoka, H., Donnelly, P. & Ward, R. High recombination rate in herpes simplex virus type 1 natural populations suggests significant co-infection1. *Infect. Genet. Evol.* **4**, 115–123 (2004).
23. Law, G. A., Herr, A. E., Cwick, J. P. & Taylor, M. P. A New Approach to Assessing HSV-1 Recombination during Intercellular Spread. *Viruses* **10**, 220 (2018).
24. Javier, R. T., Sedarati, F. & Stevens, J. G. Two Avirulent Herpes Simplex Viruses Generate Lethal Recombinants in Vivo. *Science* **234**, 746–748 (1986).
25. Hammarsten, O., Elias, P. & Stow, N. D. Characterization of a binding site for the herpes simplex virus type 1 UL9 origin-binding protein within the UL9 gene. *J. Gen. Virol.* **77**, 969–976 (1996).
26. Elias, P., Gustafsson, C. M. & Hammarsten, O. The origin binding protein of herpes simplex virus 1 binds cooperatively to the viral origin of replication oris. *J. Biol. Chem.* **265**, 17167–17173 (1990).
27. Hazuda, D. J., Perry, H. C., Naylor, A. M. & McClements, W. L. Characterization of the herpes simplex virus origin binding protein interaction with OriS. *J. Biol. Chem.* **266**, 24621–24626 (1991).
28. Wilkinson, D. & Weller, S. The Role of DNA Recombination in Herpes Simplex Virus DNA Replication. *IUBMB Life Int. Union Biochem. Mol. Biol. Life* **55**, 451–458 (2003).
29. Severini, A., Scraba, D. G. & Tyrrell, D. L. Branched structures in the intracellular DNA of herpes simplex virus type 1. *J. Virol.* **70**, 3169–3175 (1996).
30. Makhov, A. M. & Griffith, J. D. Visualization of the Annealing of Complementary Single-stranded DNA Catalyzed by the Herpes Simplex Virus Type 1 ICP8 SSB/Recombinase. *J. Mol. Biol.* **355**, 911–922 (2006).

31. Nimonkar, A. V. & Boehmer, P. E. The Herpes Simplex Virus Type-1 Single-strand DNA-binding Protein (ICP8) Promotes Strand Invasion. *J. Biol. Chem.* **278**, 9678–9682 (2003).

32. Dutch, R. E. & Lehman, I. R. Renaturation of complementary DNA strands by herpes simplex virus type 1 ICP8. *J. Virol.* **67**, 6945–6949 (1993).

33. Discipio, K. A. *et al.* Two distinct protein-protein interfaces drive cooperative binding of the herpes simplex virus protein ICP8 to ssDNA, filament formation and annealing essential for viral replication. *J. Biol. Chem.* (2025) doi:10.1016/j.jbc.2025.110498 External.

34. Nimonkar, A. V. & Boehmer, P. E. On the mechanism of strand assimilation by the herpes simplex virus type-1 single-strand DNA-binding protein (ICP8). *Nucleic Acids Res.* **31**, 5275–5281 (2003).

35. Schildgen, O., Gräper, S., Blümel, J., Külshammer, M. & Matz, B. Temperature-sensitive origin-binding protein as a tool for investigations of herpes simplex virus activities in vivo. *J. Gen. Virol.* **100**, 105–117 (2019).

36. Schildgen, O., Gräper, S., Blümel, J. & Matz, B. Genome Replication and Progeny Virion Production of Herpes Simplex Virus Type 1 Mutants with Temperature-Sensitive Lesions in the Origin-Binding Protein. *J. Virol.* **79**, 7273–7278 (2005).

37. Malik, A. K., Martinez, R., Muncy, L., Carmichael, E. P. & Weller, S. K. Genetic analysis of the herpes simplex virus type 1 UL9 gene: Isolation of a lacZ insertion mutant and expression in eukaryotic cells. *Virology* **190**, 702–715 (1992).

38. Perry, H. C., Hazuda, D. J. & McClements, W. L. The DNA Binding Domain of Herpes Simplex Virus Type 1 Origin Binding Protein Is a Transdominant Inhibitor of Virus Replication. *Virology* **193**, 73–79 (1993).

39. Stow, N. D., Hammarsten, O., Arbuckle, M. I. & Elias, P. Inhibition of Herpes Simplex Virus Type 1 DNA Replication by Mutant Forms of the Origin-Binding Protein. *Virology* **196**, 413–418 (1993).

40. Wuite, G. J. L., Smith, S. B., Young, M., Keller, D. & Bustamante, C. Single-molecule studies of the effect of template tension on T7 DNA polymerase activity. *Nature* **404**, 103–106 (2000).
41. Maier, B., Bensimon, D. & Croquette, V. Replication by a single DNA polymerase of a stretched single-stranded DNA. *Proc. Natl. Acad. Sci.* **97**, 12002–12007 (2000).
42. Morin, J. A. *et al.* Mechano-chemical kinetics of DNA replication: identification of the translocation step of a replicative DNA polymerase. *Nucleic Acids Res.* **43**, 3643–3652 (2015).
43. Andricioaei, I., Goel, A., Herschbach, D. & Karplus, M. Dependence of DNA Polymerase Replication Rate on External Forces: A Model Based on Molecular Dynamics Simulations. *Biophys. J.* **87**, 1478–1497 (2004).
44. Wang, M. D. *et al.* Force and Velocity Measured for Single Molecules of RNA Polymerase. *Science* **282**, 902–907 (1998).

REVIEWER COMMENTS

Reviewer #1 (Remarks to the Author):

I would like to congratulate the authors for their detailed and precise answers to my queries, and I therefore fully recommend this article for publication.

We thank the reviewer for their kind comment.

Reviewer #2 (Remarks to the Author):

This resubmitted manuscript entitled "HSV-1 origin binding protein UL9 forms intermolecular DNA tethers and intramolecular DNA loops" addresses most of the technical concerns raised by reviewers 2 and 3. Requested controls such as EMSA data and the inclusion of non-HSV DNA in the binding experiments were also included. However, serious problems remain regarding the presentation of background material. The introduction is still quire misleading and misses the points made in the first review. This indicates lack of understanding of the literature surrounding the mechanism of HSV DNA replication. In addition, the new description of the new lambda DNA sequences in the DNA binding experiments is not discussed accurately in the results, and their observations in the experiment are ignored in the text. Furthermore, the lack of inclusion of a UL9 mutant is troubling,

We thank the reviewer for their detailed assessment of our manuscript and hope we have addressed all of their concerns in the following sections.

Specific comments:

1. Line 45-56. "The HSV-1 genome consists of a 152 kb linear dsDNA within the viral capsid, which is believed to circularize upon entry into the host nucleus through an unknown recombination mechanism 9–14."

These references are either irrelevant to the topic of circularization during lytic infection or misleading. Papers 12-14 describe the state of the genome during latent infection, and there is no controversy about the presence of circular genomes at that stage. These new references should be removed. Paper 10 relies on the loss of HSV DNA termini during lytic infection, which is not a reliable indication as the loss of termini could occur during recombination without the formation of circular genomes.

To say that there have been reports of circularization (and theta replication) is fine, but the way this section is written implies that this model has been accepted. Reasonable doubts have been raised, the most convincing being Jackson and Deluca who have shown that during lytic infection, circular genomes are not detected. Circular genomes are only detected in the absence of the viral immediate protein ICP0. A more balanced introduction of this topic is needed. The observations that the activities of ICP0 would be expected to prevent circularization requires comment by the authors.

To emphasise that HSV-1 genomes have indeed been found in a circular conformation but that this remains controversial, as mentioned by the reviewer, we have rewritten the sentence as follows:

“The HSV-1 genome consists of a 152 kb linear dsDNA within the viral capsid, which has been found to take on a circular conformation within the host nucleus through an unknown recombination mechanism.” (Page 3, Lines 45 – 46)

As the reviewer points out, the studies 12-14 describe the presence of circular genomes during latency, which supports the sentence we cite them in. Therefore, we think it is important to still include those references.

However, we removed the Poffenberger et al. reference (previously ref. 10).

To address the reviewer’s concerns, we’ve added a description of the Jackson/DeLuca study, mentioning the role of ICP0 and added the previously requested references from the first revision:

“One study found that the HSV-1 E3 ubiquitin ligase ICP0[15,16] actively inhibits circularization during lytic infections[14]. ICP0 is well known to suppress cellular DNA damage response pathways and actively promote the degradation of DNA PK[17–22], which suggests that cellular factors promote circularization of the HSV-1 genome during latency.” (Page 3, Lines 49 – 53)

2. Lines 47-48. “However, there is evidence suggesting that HSV-1 genome circularization may not be required during lytic replication and predominantly plays a role during latency15.”

This statement is also misleading. The evidence is not that “circularization is not required”. The evidence is that circularization may not occur. This is an important distinction.

To address the reviewer’s concern, we have changed the sentence as follows:

“However, there is evidence suggesting that HSV-1 genome circularization may not occur during lytic replication and predominantly plays a role during latency.” (Page 3, Lines 47-49)

3. Lines 50-52. “The origin-dependent phase is usually depicted in a theta (θ) amplification mechanism.

Although technically correct, this is also somewhat misleading. Something like “although original models proposed that origin dependent replication occurs by a theta model, no direct evidence for this mechanism has been found and other models have been proposed” would be more appropriate.

To address the reviewer’s concern, we have changed the sentence as follows:

“Although original models proposed that origin-dependent replication occurs by a theta (θ) amplification mechanism, no direct evidence for this mechanism has been found, and other models have been proposed.” (Page 3, Lines 50-52)

4. Lines 51-52, even though no direct evidence for this mechanism has been found17,18.” This statement is correct, but incomplete and confusing. It is odd that the authors have chosen to write this paper this way, since a recombination model actually supports their own data.

This concern was addressed in 3.

5. Lines213-219, Nevertheless, we can clearly observe prolonged binding to non-origin sequences under all tested conditions. Using λ DNA as (non-HSV-1 DNA) template, we can clearly see that UL9 also binds DNA that was not derived from HSV-1 (Supplementary Fig. 4I and J). Importantly, λ-DNA contains one 216 copy of “CGTTCGCACT”, the BoxI sequence63,64, located ~20.2 kb from the center. In parallel 217 with other locations on the λ-DNA, UL9 exerts clear preference for the BoxI sequence 218 (Supplementary Fig. 4I and J).

This statement is still misleading about the data presented. Supplemental Fig 4I and 4J clearly show specific UL9 binding to three or four unique sequences along the length of the lambda genome. The authors reference the copy of BoxI sequences, but they ignore or do not comment on the other unique binding sites observed under both low and high pressure. These are partially addressed in the response to reviewer, but are essentially ignored by the author’s in the text. These data should be discussed more fully.

We agree with the reviewer that it is important to further elaborate on our observations. To address the reviewer’s concern, we have added the following sentence:

“Unfortunately, the experimental resolution and the added difficulty of two possible DNA orientations, prevent us from identifying the additional binding sequences in a more accurate manner.” (Page 10, Lines 237 – 239)

Apart from the origin sequences, there is no consistency in where UL9 binds to throughout the different conditions.

We would also like to clarify that the indicated forces are stretching forces and do not reflect pressure.

6. It is troubling that the authors have not taken the reviewers’ suggestions about using UL9 mutants. Many mutants have been expressed and purified. It is very straightforward these days to ask a company such as Azenta or others to carry out site directed mutagenesis and obtain an ATPase or DNA binding mutant. Their reasons for not doing this are not compelling. While the experiments with ATP and ATPgamma S are important, the inclusion of mutant proteins is essential to rule out that the observed activities are specific to UL9 and not a co-purifying DNA binding protein.

As suggested by the reviewer, we’ve now repeated the looping experiments with a newly purified UL9 ATP-binding mutant (E175A), which we characterised by microscale thermophoresis (MST, **Supplementary Figure 3I**). The mutant behaves like the wild-type protein in the absence of ATP, as expected, thereby supporting our initial findings. These data nicely complement our previous experiments and strongly support our hypotheses. The corresponding description and data can be found in the results section “UL9 efficiently induces oriS-independent loops on long DNA” and the Supplementary Figure 3.

“To confirm that the observed activities are specific to UL9 and not due to a contaminating DNA-binding protein, we purified and characterised an ATP-hydrolysis mutant (UL9 E175A)[67,39] (**Supplementary Figure 8**). Microscale thermophoresis (MST) confirms that this mutant cannot bind ATP (**Supplementary Figure 3I**). In the presence of ATP, the optical tweezers data show that the mutant induces 4.8 ± 2.2 (n = 17) loops per molecule, comparable to wildtype (wt) UL9 in the absence of ATP (4.7 ± 2.0, n = 33, **Figure 3**) and with shorter loop-size distribution (215 ± 69 bp, n = 81, with ATP) than the wt in the presence of ATP or non-

hydrolyzable analogue (**Supplementary Figs. 3E and F**). UL9-induced loops rupture at forces clustered around 10 pN in the absence or presence of ATP or ATPγS (**Fig. 3I-K**) or using the E175A mutant, with maximum rupture forces in the absence of ATP around 55 pN. However, addition of ATP or ATPγS reduces the maximum rupture force to below 35 pN, suggesting that ATP binding but not hydrolysis destabilizes UL9-induced loops. In the presence of ATP, the E175A mutant exhibits the highest rupture forces (~59 pN, **Supplementary Fig. 3H**), further supporting the hypothesis that ATP binding but not hydrolysis destabilises UL9-induced DNA loops (**Supplementary Fig. 3H**). Taken together, our data show that UL9 efficiently induces large oriS-independent DNA loops, which are destabilized by ATP binding." (Pages 8 and 9, Lines 194 – 212)

7. The authors were dismissive of the references and discussions about the role of ICP0. "All of these references refer to studies concerning ICP0-mediated modulation of the cellular DNA damage response, which is not directly connected to this study. While we appreciate the importance of the suggested references, we do not see a direct connection to the present study and have opted not to include them."

This is incorrect. They point out that they are not studying ICP0. What they miss here is that the presence of ICP0 has a direct correlation with the structure of the replicating DNA. Two direct lines of evidence are overlooked by the authors: (1) Jackson and Deluca only observe circular genomes in the absence of ICP0; (2) ICP0 directly degrades the essential end joining protein DNA-PK. Together these data provide evidence that during a lytic infection with ICP0, there is ample evidence that circularization of the genome would be expected to be actively inhibited.

We have addressed this concern in the specific comment 1 and included all previously requested references.

Reviewer #3 (Remarks to the Author):

I co-reviewed this manuscript with one of the reviewers who provided the listed reports. This is part of the Nature Communications initiative to facilitate training in peer review and to provide appropriate recognition for Early Career Researchers who co-review manuscripts.

Reviewer #4 (Remarks to the Author):

The authors have done an outstanding job answering the reviewers' comments. I therefore enthusiastically recommend publication.

We thank the reviewer for their kind comment.

REVIEWER COMMENTS

Reviewer #2 (Remarks to the Author):

The authors have done a good job of addressing most of the comments made in the previous reviews. I have just one last correction that needs to be made for accuracy.

We thank the reviewers for their kind words.

Line 97. “Recombination-mediated replication has emerged as a prevailing model for the initiation of HSV-1 genome replication (28,46,47), supported by extensive evidence of high levels of recombination in HSV-1 genomes in both cell culture and in vivo (31,48–52).”
It is misleading to suggest that recombination-mediated replication has emerged as a prevailing model for the initiation of HSV-1 genome replication. The models that are described in the papers cited (28, 46 and 47) actually show that recombination (specifically single strand annealing) promotes replication at existing replication forks. There is no evidence that recombination plays a role in initiation. Origin -dependent replication and recombination are considered to be distinct events in replication.

We thank the reviewers for their continued detailed assessment of our manuscript. We’ve changed the wording accordingly and added another reference (Wilkinson/ Weller, 2003) discussing the role of recombination during replication:

“Recombination has been proposed to play an intrinsic role during HSV-1 genome replication[28,46–48], supported by previous observations of the high amount of recombination of HSV-1 genomes in cell cultures and in vivo[31,49–53]. Overall, our results point to a role of UL9 during recombination but do not exclude the possibility of oriS looping-mediated initiation.”

Page 5, lines 97 - 99

Reviewer #3 (Remarks to the Author):

I co-reviewed this manuscript with one of the reviewers who provided the listed reports. This is part of the Nature Communications initiative to facilitate training in peer review and to provide appropriate recognition for Early Career Researchers who co-review manuscripts.
